# Tespa1 facilitates hematopoietic and leukemic stem cell maintenance by restricting c-Myc degradation

**DOI:** 10.1038/s41375-023-01880-6

**Published:** 2023-03-30

**Authors:** Yukai Lu, Lijing Yang, Mingqiang Shen, Zihao Zhang, Song Wang, Fang Chen, Naicheng Chen, Yang Xu, Hao Zeng, Mo Chen, Shilei Chen, Fengchao Wang, Mengjia Hu, Junping Wang

**Affiliations:** 1 State Key Laboratory of Trauma, Burns and Combined Injury, Institute of Combined Injury, Chongqing Engineering Research Center for Nanomedicine, College of Preventive Medicine, Third Military Medical University, Chongqing, 400038 China; 2 Frontier Medical Training Brigade, Third Military Medical University, Xinjiang, 831200 China; 3 Chinese PLA Center for Disease Control and Prevention, Beijing, 100071 China

**Keywords:** Acute myeloid leukaemia, Haematopoietic stem cells, Cancer stem cells

## Abstract

Hematopoietic stem cells (HSCs) and leukemia stem cells (LSCs) have robust self-renewal potential, which is responsible for sustaining normal and malignant hematopoiesis, respectively. Although considerable efforts have been made to explore the regulation of HSC and LSC maintenance, the underlying molecular mechanism remains obscure. Here, we observe that the expression of thymocyte-expressed, positive selection-associated 1 (Tespa1) is markedly increased in HSCs after stresses exposure. Of note, deletion of Tespa1 results in short-term expansion but long-term exhaustion of HSCs in mice under stress conditions due to impaired quiescence. Mechanistically, Tespa1 can interact with CSN subunit 6 (CSN6), a subunit of COP9 signalosome, to prevent ubiquitination-mediated degradation of c-Myc protein in HSCs. As a consequence, forcing c-Myc expression improves the functional defect of Tespa1-null HSCs. On the other hand, Tespa1 is identified to be highly enriched in human acute myeloid leukemia (AML) cells and is essential for AML cell growth. Furthermore, using MLL-AF9-induced AML model, we find that Tespa1 deficiency suppresses leukemogenesis and LSC maintenance. In summary, our findings reveal the important role of Tespa1 in promoting HSC and LSC maintenance and therefore provide new insights on the feasibility of hematopoietic regeneration and AML treatment.

## Introduction

Hematopoietic stem cells (HSCs) have great self-renewal and multi-lineage differentiation potential, ensuring the continuous generation of blood cells throughout lifetime [1, 2]. Under steady-state conditions, HSCs are largely dormant and usually used as a hematopoietic reservoir [3, 4]. When suffering various stresses, including irradiation, chemotherapy drug, transplantation, etc, they are able to rapidly proliferate and differentiate to produce various progeny cells [5, 6]. However, excessive activation of cell cycle may compromise HSC self-renewal and then bring about the exhaustion of HSC pool [7, 8]. Thus, a deep understanding of the mechanism of HSC maintenance may be conducive to the development of stem cell-related treatment strategy.

Acute myeloid leukemia (AML) is acknowledged as an aggressive disorder of hematologic system, with high recurrence and mortality even after receiving various treatments [9, 10]. While some therapies have been reported to delay AML progression, it is failed to effectively reduce relapse due to the low response of leukemia stem cells (LSCs) [10, 11]. LSCs are defined by immortalization and block in myeloid differentiation, which contribute to the initiation, propagation and recurrence of leukemia [12, 13]. Current research suggests that, after accumulating some genetic and epigenetic mutations, HSCs can acquire unrestrained self-renewal ability and transform into HSC counterparts in leukemia, namely LSCs [12, 14]. As a result, HSCs generally share many common properties with LSCs. So far, several regulators, such as c-Myc, STAT5B, GABPβ and Hoxa9, which have been found to play a vital role in HSC maintenance, are also involved in the regulation of LSC self-renewal [15–18]. Although considerable efforts have been made, the intrinsic molecular mechanism remains confused.

Thymocyte-expressed, positive selection-associated 1 (Tespa1), is one of the critical molecular components that regulate thymocyte development [19]. Tespa1 deficiency blocks T cell positive selection and inhibits T cell function owing to dampened TCR signaling [19, 20]. In addition, Tespa1 is reported to be involved in the regulation of the proliferation and function of B cells probably via the CD40/TRAF6 axis [21]. Recent researches show that Tespa1 also participates in the activation of mast cells, thereby modulating allergic response and airway hyperreactivity [22, 23]. These findings indicate that Tespa1 is a crucial regulator of the immune system.

In this study, we found that Tespa1 is also present in HSCs and its expression is significantly upregulated after stresses. Loss of Tespa1 resulted in short-term expansion but long-term exhaustion of HSCs during hematopoietic stresses. Mechanism studies revealed that Tespa1 regulates CSN subunit 6 (CSN6)-mediated c-Myc pathway which is required to maintain HSC quiescence and function. Additionally, we identified that Tespa1 can serve as a pro-oncogenic factor both in human and murine AML by maintaining LSC stemness. Collectively, our study uncovers a previously unrecognized function of Tespa1 in HSC and LSC biology and offers new avenues for long-term hematopoietic maintenance and clinical treatment of AML.

## Materials and methods

### Mice

B6.129S-Tespa1^tm1Smoc^ (*Tespa1*^*-/-*^) mice were purchased from the Model Organisms Center (Shanghai, China) and backcrossed to C57BL/6 background for more than 6 generations. Normal C57BL/6 mice were obtained from the Institute of Zoology (Chinese Academy of Sciences, Beijing, China). B6. SJL (CD45.1) mice were generously given by Prof. Jinyong Wang (Guangzhou Institutes of Biomedicine and Health, Chinese Academy of Science, Guangzhou, China). Mice were male and analyzed at 8–10 weeks old except for investigating middle aged mice (12 months old). Mice were randomly allocated and sample number was estimated based on extensive experience. No blinding was conducted and no samples were excluded in our study. All mice were housed in our lab and all experimental procedures were approved by the Animal Care Committee of Third Military Medical University (Chongqing, China).

### AML patient and healthy donor samples

All BM specimens from AML patient and healthy donor were obtained from Southwest Hospital and Daping Hospital (The first and third affiliated hospitals of Third Military Medical University) with informed consent. All studies involving human samples were approved by the Ethics Committee of Third Military Medical University.

### Flow cytometry analysis and cell sorting

Mouse BM and PB samples were prepared as we described [8, 24]. To analyze the phenotype of normal hematopoietic cells, samples were stained with anti-lineage cocktail (anti-CD3e, anti-CD11b, anti-Gr-1, anti-B220 and anti-Ter-119), anti-Sca-1, anti-c-Kit, anti-CD34, anti-Flk2, anti-CD150, anti-CD48, anti-CD127, anti-CD16/32, anti-Gr-1, anti-CD11b, anti-B220, anti-CD3e, anti-CD45.1 and anti-CD45.2. To analyze the phenotype of LSCs, anti-lineage cocktail, anti-Sca-1, anti-c-Kit, anti-CD34 and anti-CD16/32 were used. The apoptosis, cell cycle, in vivo BrdU incorporation and intracellular staining assays were performed following our previous protocols [24]. Flow cytometry analysis was conducted using a FACSVerse (BD Biosciences, San Jose, CA, USA) or ID7000 (Sony Biotechnology, Tokyo, Japan). For cell sorting, a Direct Lineage Cell Depletion Kit (Miltenyi Biotec, Bergisch Gladbach, Germany) was used to delete the mature cells and then samples were stained with above antibodies, followed by sorting using a FACSAria III (BD Biosciences). Gating strategies are referred to our previous studies [8, 24]. The details of antibodies are provided in Supplementary Table S1.

### Lentivirus infection

All lentivirus were produced by Tsingke Biotechnology (Beijing, China). For TESPA1 knockdown, the target sequences for shRNA were provided in Supplementary Table S2. HEL, MOLM-13 cells and primary human AML cells were infected by the concentrated virus in the presence of 4 μg/ml polybrene (Tsingke) for 48 h, and then positive infected cells were sorted for subsequent experiments. For overexpression of CSN6 and c-Myc, sorted LSKs were transfected as we described [25]. Forty-eight hours later, GFP-positive cells were sorted for subsequent experiments.

### Murine MLL-AF9 leukemia model

This assay was performed as previously described [26]. MSCV-MLL-AF9-IRES-GFP vectors were transfected into HEK293T cells with packaging plasmids pKat and VSVG (Tsingke) using DNA transfection reagent (Neofect Biotech, Beijing, China). At 48 and 72 hours after transfection, viral supernatants were collected and filtered. For generation of leukemia cells, Lin^-^ cells isolated from *Tespa1*^*+/+*^ and *Tespa1*^*-/-*^ mice were spin-infected with supernatants containing MLL-AF9 retroviruses twice supplemented with 50 ng/ml SCF (PeproTech, Rocky Hill, NJ, USA), 10 ng/ml IL-3 (PeproTech), 10 ng/ml IL-6 (PeproTech) and 8 μg/ml polybrene (Tsingke). GFP-positive cells (pre-leukemic cells) were sorted and intravenously transplanted into 7.5 Gy irradiated normal recipient mice.

### Statistical analysis

The experimental data were analyzed using GraphPad Prism 6.0 (GraphPad Software, La Jolla, CA, USA) and presented as mean±SD. Variance was similar among the groups. Significance testing between two groups were performed by two-tailed Student’s *t*-test and that among multiple groups were analyzed using one-way analysis of variance (ANOVA). Kaplan-Meier survival curves were compared with the log-rank test. All experiments were independently performed at least three times. ^*^P < 0.05, ^**^P < 0.01 were considered statistically significant.

## Results

### Tespa1 is dispensable for normal hematopoiesis but its deficiency facilitates HSC expansion upon short-term stress

Tespa1 was initially defined as a thymus specifically expressed gene [19]. Indeed, in addition to thymus, we found that Tespa1 also expressed in HSCs by analyzing the BioGPS database, which was further confirmed by qPCR analysis (Supplementary Fig. S1A, B). Then, we generated Tespa1 knockout (*Tespa1*^*-/-*^) mice but found that loss of Tespa1 caused no overt alteration in total BM cellularity and PB counts (Supplementary Fig. S1C, D). Flow cytometric analysis also revealed that the frequencies and numbers of HSCs, committed-progenitor cells, and mature cells (except for T cells) were comparable between *Tespa1*^*+/+*^ and *Tespa1*^*-/-*^ BM (Supplementary Fig. S1E, F). Thus, Tespa1 may be not essential for homeostatic hematopoiesis in mice.

**Fig. 1:**
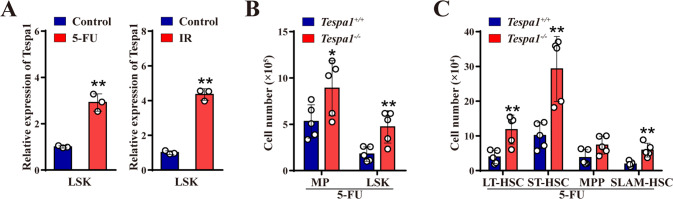
Thymocyte-expressed, positive selection-associated 1 (Tespa1) deficiency facilitates hematopoietic stem cell (HSC) expansion upon short-term stress. **A** Quantitative real-time PCR (qPCR) analysis of Tespa1 mRNA expression in LSKs isolated from mice (left) at day 9 after 5-fluorouracil (5-FU) injection or (right) at day 13 after irradiation (IR) exposure (*n* = 3). Untreated mice were served as controls. LSK, Lin^-^ Sca1^+^ c-Kit^+^. **B**, **C** The numbers (two femurs and tibias) of myeloid progenitors (MPs), LSKs, long-term HSCs (LT-HSCs), short-term HSCs (ST-HSCs), multipotent progenitors (MPPs) and signaling lymphocytic activation molecule (SLAM)-HSCs in the bone marrow (BM) of *Tespa1*^*+/+*^ and *Tespa1*^*-/-*^ mice at day 9 following 5-FU injection (*n* = 5). MP, Lin^-^ Sca1^-^ c-Kit^+^; LT-HSC, Lin^-^ Sca1^+^ c-Kit^+^ CD34^-^ Flk2^-^; ST-HSC, Lin^-^ Sca1^+^ c-Kit^+^ CD34^+^ Flk2^-^; MPP, Lin^-^ Sca1^+^ c-Kit^+^ CD34^+^ Flk2^+^; SLAM-HSC, Lin^-^ Sca1^+^ c-Kit^+^ CD150^+^ CD48^-^. ^*^P < 0.05, ^**^P < 0.01.

Interestingly, we discovered that Tespa1 was significantly upregulated in LSKs (Lin^−^ Sca-1^+^ c-Kit^+^) following 5-fluorouracil (5-FU) and irradiation (IR) treatment (Fig. 1A), hinting that Tespa1 may regulate hematopoiesis under stress conditions. As anticipated, compared with controls, the frequencies and numbers of LSKs, long-term HSCs (LT-HSCs), short-term HSCs (ST-HSCs), and SLAM-HSCs (identified by signaling lymphocytic activation molecules) were increased in *Tespa1*^-/-^ BM at day 9 post 5-FU treatment (Fig. 1B, C; Supplementary Fig. S2A). On the other hand, overall similar trend of HSPC changes were observed in *Tespa1*^-/-^ mice when subjected to 5.0 Gy total body irradiation (Supplementary Fig. S2B, C). Consistent with these results, significantly faster recovery of white blood cell (WBC) and platelet (PLT) were also observed in *Tespa1*^-/-^ mice after 5-FU and IR treatment (Supplementary Fig. S2D, E). Of note, expanded percentages and numbers of megakaryocyte/erythroid progenitors (MEPs) and common lymphoid progenitors (CLPs) were found in the BM from *Tespa1*^-/-^ mice with 5-FU treatment (Supplementary Fig. S2F). In contrast, we observed increased percentages and numbers of granulocyte/macrophage progenitors (GMPs) and common myeloid progenitors (CMPs) in *Tespa1-*deleted BM following IR exposure (Supplementary Fig. S2G). Taken together, our data suggest that Tespa1 ablation promotes short-term HSC expansion under hematopoietic stresses.

### Loss of Tespa1 impairs the long-term HSC maintenance

**Fig. 2:**
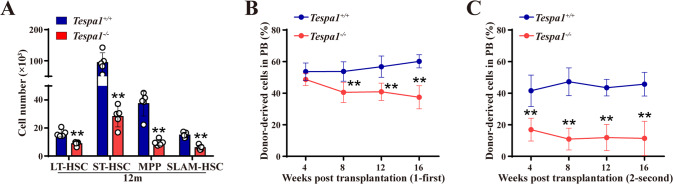
Loss of Tespa1 impairs the long-term HSC maintenance. **A** The numbers (two femurs and tibias) of indicated populations in the BM of 12-month (m)-old *Tespa1*^*+/+*^ and *Tespa1*^*-/-*^ mice (*n* = 5). **B**, **C** The percentages of donor-derived cells in recipients’ peripheral blood (PB) at the indicated time points after first and second BM transplantation (BMT) (*n* = 6–8). ^**^P <  0.01.

To evaluate whether Tespa1 deletion influences long-term HSC maintenance, we then analyzed the hematopoietic phenotypes in middle-aged *Tespa1*^*+/+*^ and *Tespa1*^*-/-*^ mice. Despite the comparable BM cellularity, the frequencies and numbers of LSK and subpopulations were significantly reduced after Tespa1 ablation (Fig. 2A; Supplementary Fig. S3A–C). Besides, we observed an increased frequency and number of CMPs but a decreased frequency and number of GMPs and CLPs in 12-month-old *Tespa1*^*-/-*^mice (Supplementary Fig. S3D).

To further determine this finding, we performed a competitive BM transplantation (BMT) assay (Supplementary Fig. S4A). It was noticed that donor-derived cells were significantly reduced in recipients after first and second transplantation when Tespa1 was deleted (Fig. 2B, C; Supplementary Fig. S4B). Meanwhile, the multi-lineage reconstitution of *Tespa1*^*-/-*^ cells was dramatically decreased post-transplant (Supplementary Fig. S4C). Moreover, recipient mice transplanted with *Tespa1*^*-/-*^ cells displayed a remarkable reduction in chimeric levels of BM cells, MPs, and HSCs compared with that receiving transplants from *Tespa1*^*+/+*^ cells (Supplementary Fig. S4D). To strengthen our findings, we then conducted a competitive HSC transplantation (HSCT) assay (Supplementary Fig. S4E). Actually, similar results were obtained after first and second transplantation (Supplementary Fig. S4F–H). Next, we performed reciprocal BM transplantation and found that the chimerism levels were comparable between the two groups of recipients (Supplementary Fig. S5A, B). In addition, we did not observe any significant difference in the homing ability between *Tespa1*^*+/+*^and *Tespa1*^*-/-*^ HSCs (Supplementary Fig. S5C, D). Altogether, these findings indicate that Tespa1 maintains the long-term self-renewal capacity of HSCs in a cell-intrinsic manner.

### Tespa1 deletion decreases HSC quiescence during hematopoietic stress

**Fig. 3:**
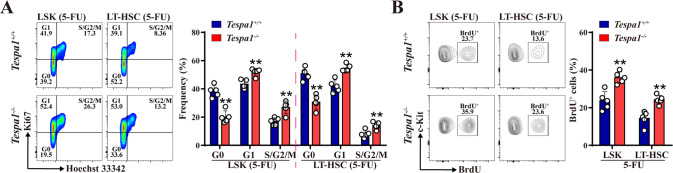
Tespa1 deletion decreases HSC quiescence during hematopoietic stress. **A** Flow cytometric analysis of the cell cycle of LSKs and LT-HSCs in the BM of *Tespa1*^*+/+*^ and *Tespa1*^*-/-*^ mice at day 9 after 5-FU injection (*n* = 5). **B** Flow cytometric analysis of the proportion of bromodeoxyuridine (BrdU)^+^ cells in LSKs and LT-HSCs from *Tespa1*^*+/+*^ and *Tespa1*^*-/-*^ mice at day 9 following 5-FU injection (*n* = 5). ^**^P < 0.01.

In view of the observations that Tespa1 deficiency causes short-term expansion but long-term exhaustion of HSCs during hematopoietic stress, we speculated that Tespa1 may modulate HSC quiescence. However, no obvious alteration was observed in the cell cycle distribution in LSKs and LT-HSCs after ablation of Tespa1 under steady-state conditions (Supplementary Fig. S6A). Intriguingly, *Tespa1*^-/-^ HSCs had a reduced percentage in G0 phase but an increased percentage in G1 and S/G2/M phase after 5-FU treatment (Fig. 3A). Similar results were detected in SLAM-HSCs in the absence of Tespa1 (Supplementary Fig. S6B, C). Furthermore, in vivo BrdU incorporation assay revealed that deletion of Tespa1 increased the proliferation of HSCs in mice following 5-FU challenge (Fig. 3B; Supplementary Fig. S6D). Consistent with these data, Tespa1 deficiency accelerated HSC proliferation after IR (Supplementary Fig. S6E–G). However, apoptosis rates were comparable between *Tespa1*^+/+^ and *Tespa1*^-/-^ HSCs (Supplementary Fig. S6H-J). These findings suggest that Tespa1 inhibits the excessive activation of HSCs in response to stress stimuli.

### Tespa1 knockout leads to reduced c-Myc signaling in HSCs upon stress

**Fig. 4:**
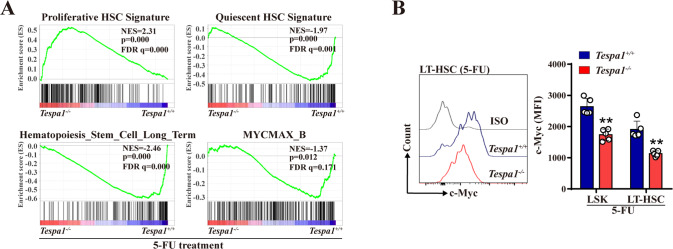
Tespa1 knockout leads to reduced c-Myc signaling in HSCs upon stress. **A** Gene set enrichment analysis (GSEA) of RNA-sequencing (RNA-seq) data with HSC-related gene sets and MYC-related gene set at day 9 following 5-FU injection. **B** Flow cytometric analysis of c-Myc protein expression in LSKs and LT-HSCs from *Tespa1*^*+/+*^ and *Tespa1*^*-/-*^ mice at day 9 following 5-FU injection (*n* = 5). ^**^P < 0.01.

To elucidate the underlying molecular mechanisms that Tespa1 orchestrates HSC response to stresses, we conducted RNA-sequencing (RNA-seq) analysis using *Tespa1*^+/+^ and *Tespa1*^-/-^ LSKs upon 5-FU and IR treatment. It was found that 1093 genes were upregulated and 896 genes were downregulated in *Tespa1* KO LSKs relative to controls after 5-FU treatment (Supplementary Fig. S7A–C). Meanwhile, 1446 genes were upregulated and 2336 genes were downregulated in LSKs after Tespa1 deletion when exposed to IR (Supplementary Fig. S7A–C). Subsequently, we identified that 194 upregulated genes and 261 downregulated genes were overlapped in *Tespa1*^*-/-*^ HSCs following 5-FU and IR exposure (Supplementary Fig. S7A). GSEA disclosed that HSC proliferative signature was enriched in *Tespa1*^*-/-*^ HSCs, while stemness-related signature, quiescent signature were enriched in *Tespa1*^*+/+*^ HSCs (Fig. 4A; Supplementary Fig. S7D, E), further consolidating our above results.

In particular, we noted that MYC signature was downregulated in HSCs with Tespa1 deficiency (Fig. 4A; Supplementary Fig. S7E). Previous studies have indicated that c-Myc is vital for preserving the quiescence and long-term reconstitution function of HSCs [27–29]. Indeed, the expression of c-Myc target genes associated with HSC maintenance, including *Myct1*, *Jmjd1c*, *Nr4a1*, *Nr4a2, Nr4a3* and *Egr1*, was significantly decreased in Tespa1-deficient HSCs upon stresses, which was proved by qPCR (Supplementary Fig. S7B, C, F, G). Surprisingly, c-Myc mRNA expression was largely unchanged in HSCs in the absence of Tespa1 (Supplementary Fig. S7H). However, Tespa1 deficiency drastically reduced the protein level of c-Myc in HSCs (Fig. 4B; Supplementary Fig. S7I). Hence, these results suggest that Tespa1 may sustain c-Myc protein stability in HSCs during hematopoietic stresses.

### Tespa1 interacts with CSN6 to inhibit c-Myc degradation in HSCs

**Fig. 5:**
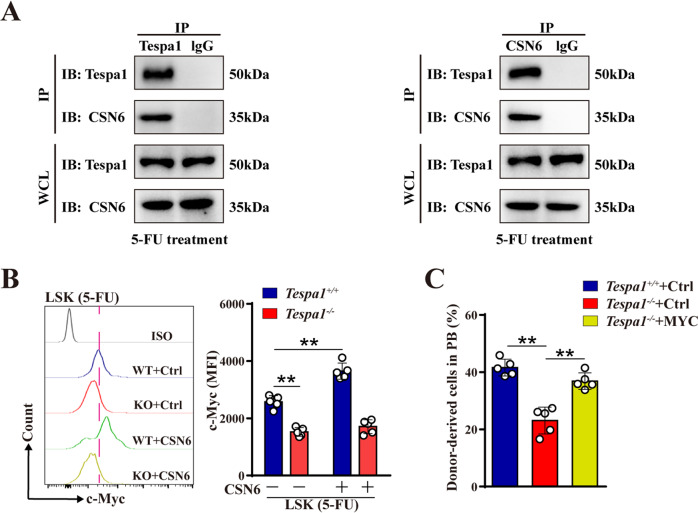
Tespa1 interacts with CSN subunit 6 (CSN6) to inhibit c-Myc degradation in HSCs. **A** Co-immunoprecipitation analysis of Tespa1 and CSN6 interaction in Lin^-^ c-Kit^+^ cells purified from the BM of mice at day 9 after 5-FU injection. IgG antibody was used as the negative control. IP, immunoprecipitation; WCL, whole cell lysate. **B** LSKs from the BM of *Tespa1*^*+/+*^ and *Tespa1*^*-/-*^ mice at day 9 post 5-FU injection were transduced with control (Ctrl) or CSN6 overexpression lentivirus. Then, c-Myc protein expression in LSKs was analyzed by flow cytometry after transduction (*n* = 5). **C** LSKs from *Tespa1*^*+/+*^ and *Tespa1*^*-/-*^ BM were transduced with control (Ctrl) or c-Myc overexpression lentivirus. Green fluorescent protein (GFP)^+^ cells were then transplanted into CD45.1 recipient mice with CD45.1 competitor BM cells. The percentage of donor-derived cells in PB of recipient mice at 16 weeks after transplantation (*n* = 5). ^**^P  < 0.01.

It has been established that c-Myc protein is mainly degraded by the ubiquitin-proteasome system [30, 31]. Notably, CSN6, a subunit of COP9 signalosome, was reported to stabilize c-Myc protein by decreasing its ubiquitination [32, 33]. Given that CSN6 is a potential Tespa1 interacting factor by protein-protein interaction analysis using STRING database (Supplementary Fig. S8A), we first conducted an in situ proximity ligation assay and found that Tespa1 could interact with CSN6 in HSCs upon 5-FU and IR treatment (Supplementary Fig. S8B, C). This notion was further confirmed by immunofluorescence co-localization analysis and co-immunoprecipitation assays (Fig. 5A; Supplementary Fig. S8D–F). Expectedly, both mRNA and protein levels of CSN6 were comparable between *Tespa1*^*+/+*^ and *Tespa1*^*-/-*^ HSCs (Supplementary Fig. S9A–C). Hence, we assumed that the reduced protein level of c-Myc in *Tespa1*^*-/-*^ HSCs may be attributed to the alteration of CSN6 function. To validate this conjecture, CSN6 was overexpressed in LSKs in the context of 5-FU treatment and we found that enforcing CSN6 expression increased the protein level of c-Myc in *Tespa1*^+/+^ HSCs but not *Tespa1*^*-/-*^ cells, although c-Myc mRNA level was not altered (Fig. 5B; Supplementary Fig. S9D). On the other hand, treatment with MG-132, a proteasome inhibitor, substantially elevated the protein level of c-Myc in *Tespa1*^*-/-*^ HSCs (Supplementary Fig. S9E). These data illustrate that Tespa1 can interact with CSN6 to inhibit c-Myc degradation in HSCs during hematopoietic stresses.

Next, to determine whether the defective quiescence and function of Tespa1-null HSCs is driven by the attenuation of c-Myc signaling, *Tespa1*^*-/-*^ HSCs were overexpressed with c-Myc (Supplementary Fig. S9F). Consequently, c-Myc overexpression inhibited abnormal proliferation and rescued the functional defect of HSCs in the absence of Tespa1 (Fig. 5C; Supplementary Fig. S9G, H). Overall, our data indicate that Tespa1 regulates HSC maintenance via the CSN6/c-Myc axis.

### TESPA1 is essential for human AML cell growth

**Fig. 6:**
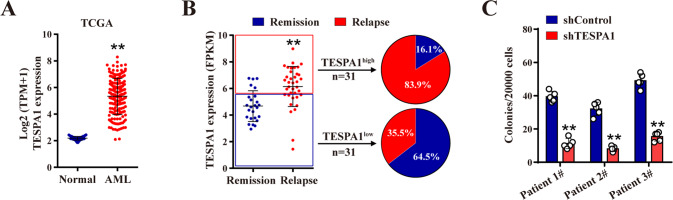
TESPA1 is essential for human acute myeloid leukemia (AML) cell growth. **A** The expression of TESPA1 in AML patients and healthy donors. The data (173 AML patients and 70 healthy donors) were derived from The Cancer Genome Atlas (TCGA) database. **B** Distribution of TESPA1 expression in AML patients with remission or relapse. Data were obtained from Vizome database (http://www.vizome.org/). **C** The colony formation assays of primary human AML cells after TESPA1 knockdown. Each dot represents one culture. ^**^P <  0.01.

AML cells are characterized by incontrollable growth due to the disordered cell cycle [13]. To investigate whether TESPA1 is also involved in the regulation of AML biology, we first analyzed its expression in AML samples and healthy controls from The Cancer Genome Atlas (TCGA) and Gene expression Ominibus (GEO) databases. A significantly elevated expression of TESPA1 was observed in AML cells in comparation with controls, which was confirmed in primary AML specimens by qPCR analysis (Fig. 6A; Supplementary Fig. S10A, B). In addition, human single-cell data from Atlas of Blood Cells database exhibited that malignant hematopoietic cells had a higher level of TESPA1 than non-malignant ones (Supplementary Fig. S10C). Consistently, receiver-operating characteristic (ROC) curve showed a good diagnostic performance of TESPA1 for AML (Supplementary Fig. S10D). Meanwhile, prognostic evaluation using the data from public databases showed that high expression of TESPA1 was associated with increased relapse percentage and decreased overall survival of AML patients (Fig. 6B; Supplementary Fig. S10E). These data imply that TESPA1 may play a potential role in AML progression. To further confirm this possibility, we measured human AML cell lines and then knocked down TESPA1 expression in HEL and MOLM-13 cells (Supplementary Fig. S10F, G). Knockdown of TESPA1 inhibited cell growth and colony formation both in HEL and MOLM-13 cells (Supplementary Fig. S10H, I), with no obvious alteration of apoptosis rate (Supplementary Fig. S10J, K). More importantly, TESPA1 knockdown also significantly suppressed primary human AML cell growth (Fig. 6C; Supplementary Fig. S10L). In accordance with above data, TESPA1 knockdown did not affect mRNA level but reduced protein expression of c-Myc in AML cells (Supplementary Fig. S10M-O). Taken together, TESPA1 may contribute to the occurrence and development of AML.

### Tespa1 drives murine AML progression and maintains LSC function

**Fig. 7:**
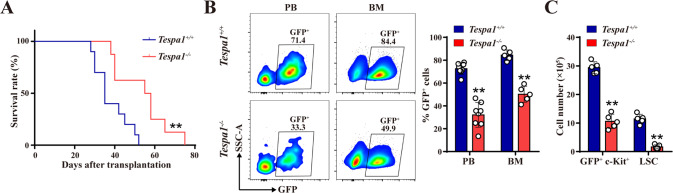
Tespa1 drives murine AML progression and maintains leukemia stem cell (LSC) function. **A** The survival curves for mice after transplanted with *Tespa1*^*+/+*^ and *Tespa1*^*-/-*^ pre-leukemic cells (*n* = 8–10). **B** Flow cytometric analysis of the percentage of GFP^+^ cells in the PB and BM from primary AML mice at day 25 after transplantation (*n* = 5–8). **C** The numbers (two femurs and tibias) of GFP^+^ c-Kit^+^ cells and LSCs in the BM from recipients at day 25 after transplantation (*n* = 5). LSC, Lin^-^ Sca1^-^ GFP^+^ c-Kit^+^ CD34^+^ CD16/32^+^. ^**^P < 0.01.

To comprehensively understand the role of Tespa1 in leukemogenesis, we employed the MLL-AF9-induced murine AML model (Supplementary Fig. S11A). Tespa1 deletion substantially reduced the colony forming ability of pre-leukemic cells in vitro and prolonged AML mice survival (Fig. 7A; Supplementary Fig. S11B). Furthermore, we observed that *Tespa1*^*-/-*^ AML mice exhibited a largely reduced ratio of GFP^+^ leukemic cells in the PB and BM, accompanied by decreases in malignant cells and total WBC count in the PB (Fig. 7B; Supplementary Fig. S11C, D). Not surprisingly, spleen, lung and liver showed lower weights and less immature cell infiltration in recipients transplanted with *Tespa1*^*-/-*^ AML cells in comparation with controls (Supplementary Fig. S11E, F).

Finally, we analyzed the characteristics of LSCs in murine AML model, and found that the percentages and numbers of GFP^+^ c-Kit^+^ cells and LSCs were evidently reduced after Tespa1 deletion (Fig. 7C; Supplementary Fig. S11G). However, comparable apoptosis rates of GFP^+^ c-Kit^+^ cells were detected in *Tespa1*^*+/+*^ and *Tespa1*^*-/-*^ AML mice (Supplementary Fig. S11H). Notably, we also observed a prominent reduction in c-Myc expression in Tespa1-deficient LSCs relative to controls (Supplementary Fig. S11I). Collectively, we reasonably conclude that Tespa1 plays a crucial role in promoting the maintenance of LSCs.

## Discussion

HSCs contribute to the hematopoietic homeostasis under normal physiological conditions and mediate hematopoietic regeneration after stresses, while LSCs drive the initiation, progression and recurrence of AML [1, 5, 12]. They share some common characteristics, to some extent, both having intensive self-renewal potential [13, 17]. Despite increasing attention has been focused on the regulatory network of both stem cells, a deep understanding is still lacking. Here, our study shows for the first time that Tespa1 preserves the self-renewal of HSCs and LSCs through supporting c-Myc signaling.

It was reported that Tespa1, as a gene specifically expressed in thymus, plays an important role in T cell development [19, 20]. In our study, we first observed that Tespa1 is also present in HSCs and is remarkably elevated when exposed to 5-FU and IR. Then, using *Tespa1*^*-/*-^ mice, we found that although Tespa1 is dispensable for homeostatic hematopoiesis, its ablation accelerated hematopoietic recovery during short-term stresses, suggesting that stress stimuli might amplify the role of Tespa1 in HSCs. Consistently, *Tespa1*^*-/*-^ HSCs exhibited significantly reduced quiescence and increased proliferation after stresses. However, the changes of some hematopoietic phenotypes were not completely uniform following exposure to 5-FU and IR, which might be due to the different action mechanisms of these two stress factors. It was well accepted that quiescence maintenance is vital for preserving the self-renewal capacity of HSCs and preventing them from exhaustion during continuous hematopoietic output [7, 8]. Numerous studies, including our own work, have shown that HSC proliferation is needed for hematopoietic recovery, whereas excessive amplification of HSC number may consume their self-renewal ability, eventually leading to hematopoietic failure [24, 34]. In line with this notion, transplantation assays confirmed that the long-term self-renewal capacity of HSCs were severely compromised in the absence of Tespa1. Therefore, our findings manifest that Tespa1 is necessary for the maintenance of HSC stemness and function.

c-Myc is a well-known signaling molecule that is implicated in multiple biological process, including cell metabolism, stemness maintenance, cell cycle regulation, etc [35, 36]. In recent years, the role of c-Myc in HSCs has been a topic of debate. Some researchers hold the opinion that c-Myc is a proliferation-promoting gene and its overexpression decreases HSC self-renewal [37, 38]. However, others point out that c-Myc promotes the maintenance of HSC quiescence and function, because it can directly control the transcription of several stemness-associated genes [27–29]. In this work, we found that deletion of Tespa1 results in a reduced protein level of c-Myc in HSCs during hematopoietic regeneration, accompanied by impaired HSC quiescence and long-term repopulating function. Additionally, we discovered that c-Myc target genes that have been shown to promote HSC maintenance, including *Myct1*, *Jmjd1c*, *Nr4a1*, *Nr4a2*, *Nr4a3* and *Egr1* [39–43], were obviously downregulated in Tespa1-ablated HSCs after stresses. These observations are similar to those seen in mice with haploinsufficiency of c-Myc [27]. Importantly, overexpression of c-Myc improved the defects of *Tespa1*^*-/-*^ HSCs, indicating that the effect of Tespa1 on HSCs, at least in part, via c-Myc. On the other hand, there are some studies concerning the role of c-Myc on HSC apoptosis, but we did not detect any appreciable change in *Tespa1*^*+/+*^ and *Tespa1*^*-/-*^ HSCs upon stresses in our experiments. A potential explanation for this discordance is the compensatory effect of c-Myc target genes on HSC apoptosis, as previously reported [39, 42]. Collectively, our data disclose that Tespa1 facilitates HSC maintenance probably through sustaining c-Myc signaling during stress hematopoiesis.

It is worth that Tespa1 was previously discovered to orchestrate the TCR pathway in T cells by interacting with IP3R, PLC-γ1 and Grb2 [19], which suggests that Tespa1 is an important signal regulatory factor. Accumulating evidence reveals that COP9 signalosome, an evolutionary conserved protein complex, is involved in suppressing the ubiquitin-proteasome system that mediates the degradation of intracellular proteins [32, 44]. CSN6 is a subunit of COP9 signalosome and has been shown to enhance the stability of some proteins, such as c-Myc, FOXO4 and EGFR, by decreasing their ubiquitination [33, 45, 46]. A previous investigation reported that overexpression of CSN5, a homologue of CSN6, leads to HSC proliferation and development of a myeloproliferative disorder in mice by increasing p53 degradation [47]. Here, we noticed that there is an interaction between Tespa1 and CSN6 protein and Tespa1 deficiency leads to reduced protein levels of c-Myc rather than CSN6 in HSCs. Subsequently, treatment with MG-132, a proteasome inhibitor which has been reported to repress c-Myc degradation [48, 49], virtually restored c-Myc level in *Tespa1*^*-/-*^ HSCs. More importantly, overexpression of CSN6 increased c-Myc protein level in *Tespa1*^*+/+*^ HSCs but not in *Tespa1*^*-/-*^ HSCs, indicating that CSN6 stabilizes c-Myc in the presence of Tespa1. Accordingly, it is reasonable to propose that Tespa1 can interact with CSN6 to inhibit c-Myc degradation in HSCs.

AML is a hematopoietic malignancy, which arises from uncontrolled expansion and blocked differentiation of LSCs [12, 50]. In addition to regulating normal hematopoiesis, c-Myc also has a pro-oncogenic role in AML [51]. Deletion of c-Myc can evidently reduce LSC self-renewal and survival [15, 52]. It is reasonable because many well recognized stemness genes are proto-oncogenes, such as Meis1, Msi2, Hoxa9 and Evil [53–56]. In the present study, we found that Tespa1 is remarkably upregulated in human AML cells and its deletion significantly suppresses the growth of AML cells. Additionally, we discovered that Tespa1 ablation inhibits AML progression and improves the survival of mice with MLL-AF9-induced AML. More importantly, the frequency and number of LSCs was decreased when Tespa1 was ablated, along with a reduction in c-Myc protein expression. These data indicate that Tespa1 facilitates AML progression and LSC functional maintenance probably through reducing c-Myc degradation. Furthermore, data from cBioPortal and Vizome databases showed that no mutation of TESPA1 was present in patients with AML and no more than 0.1% mutations of TESPA1 in patients with other types of leukemia [57, 58], suggesting that TESPA1 may be a relatively stable target for clinical treatment of AML. On the other hand, considering that Tespa1 is dispensable for homeostatic hematopoiesis, it is possible to explore potential Tespa1 inhibitors that can selectively target quiescent LSCs without apparent toxicity to normal HSCs in AML patients, similar to previous reports [59, 60]. Notably, Tespa1 inhibitors should be used with caution to protect the function of normal HSCs in AML patients exposed to stress stimuli. After all, stress may upregulate the expression of Tespa1 in normal HSCs and Tespa1 deficiency may affect hematopoiesis under stress conditions, as shown in our study.

In conclusion, we demonstrate that loss of Tespa1 in HSCs and LSCs reduces c-Myc protein stability, impairing stress hematopoiesis and AML propagation. Consequently, our data uncover a key role of Tespa1 in regulating the maintenance of HSCs and LSCs and provide a new target for hematopoietic regeneration and AML treatment.

## Supplementary information

Supplementary Information

Supplementary Table S1

Supplementary Table S2

## Data Availability

The raw data of RNA-seq were deposited in NCBI GEO database with accession number GSE211292 and GSE211117.

## References

[1] Eaves CJ (2015). Hematopoietic stem cells: concepts, definitions, and the new reality. Blood..

[2] Laurenti E, Gottgens B (2018). From haematopoietic stem cells to complex differentiation landscapes. Nature..

[3] Cabezas-Wallscheid N, Buettner F, Sommerkamp P, Klimmeck D, Ladel L, Thalheimer FB (2017). Vitamin A-Retinoic Acid Signaling Regulates Hematopoietic Stem Cell Dormancy. Cell..

[4] Nakamura-Ishizu A, Takizawa H, Suda T (2014). The analysis, roles and regulation of quiescence in hematopoietic stem cells. Development..

[5] Wilson A, Laurenti E, Oser G, van der Wath RC, Blanco-Bose W, Jaworski M (2008). Hematopoietic stem cells reversibly switch from dormancy to self-renewal during homeostasis and repair. Cell..

[6] Pietras EM, Reynaud D, Kang YA, Carlin D, Calero-Nieto FJ, Leavitt AD (2015). Functionally Distinct Subsets of Lineage-Biased Multipotent Progenitors Control Blood Production in Normal and Regenerative Conditions. Cell Stem Cell.

[7] Wang N, Yin J, You N, Yang S, Guo D, Zhao Y (2021). TWIST1 preserves hematopoietic stem cell function via the CACNA1B/Ca2+/mitochondria axis. Blood..

[8] Hu M, Zeng H, Chen S, Xu Y, Wang S, Tang Y (2018). SRC-3 is involved in maintaining hematopoietic stem cell quiescence by regulation of mitochondrial metabolism in mice. Blood..

[9] Yi M, Li A, Zhou L, Chu Q, Song Y, Wu K. The global burden and attributable risk factor analysis of acute myeloid leukemia in 195 countries and territories from 1990 to 2017: estimates based on the global burden of disease study 2017. J Hematol Oncol. 2020;13:72. 10.1186/s13045-020-00908-z PMC7282046 32513227

[10] Shlush LI, Mitchell A, Heisler L, Abelson S, Ng SWK, Trotman-Grant A (2017). Tracing the origins of relapse in acute myeloid leukaemia to stem cells. Nature..

[11] Rautenberg C, Germing U, Haas R, Kobbe G, Schroeder T. Relapse of Acute Myeloid Leukemia after Allogeneic Stem Cell Transplantation: Prevention, Detection, and Treatment. Int J Mol Sci. 2019;20:228. 10.3390/ijms20010228 PMC6337734 30626126

[12] Vetrie D, Helgason GV, Copland M (2020). The leukaemia stem cell: similarities, differences and clinical prospects in CML and AML. Nat Rev Cancer.

[13] O’Reilly E, Zeinabad HA, Szegezdi E (2021). Hematopoietic versus leukemic stem cell quiescence: Challenges and therapeutic opportunities. Blood Rev.

[14] Huntly BJ, Gilliland DG (2005). Leukaemia stem cells and the evolution of cancer-stem-cell research. Nat Rev Cancer.

[15] Amaya ML, Inguva A, Pei S, Jones C, Krug A, Ye H (2022). The STAT3-MYC axis promotes survival of leukemia stem cells by regulating SLC1A5 and oxidative phosphorylation. Blood..

[16] Kollmann S, Grausenburger R, Klampfl T, Prchal-Murphy M, Bastl K, Pisa H (2021). A STAT5B-CD9 axis determines self-renewal in hematopoietic and leukemic stem cells. Blood..

[17] Yu S, Jing X, Colgan John D, Zhao D-M, Xue H-H (2012). Targeting Tetramer-Forming GABPβ Isoforms Impairs Self-Renewal of Hematopoietic and Leukemic Stem Cells. Cell Stem Cell.

[18] Smith LL, Yeung J, Zeisig BB, Popov N, Huijbers I, Barnes J (2011). Functional crosstalk between Bmi1 and MLL/Hoxa9 axis in establishment of normal hematopoietic and leukemic stem cells. Cell Stem Cell.

[19] Wang D, Zheng M, Lei L, Ji J, Yao Y, Qiu Y (2012). Tespa1 is involved in late thymocyte development through the regulation of TCR-mediated signaling. Nat Immunol.

[20] Liang J, Lyu J, Zhao M, Li D, Zheng M, Fang Y, et al. Tespa1 regulates T cell receptor-induced calcium signals by recruiting inositol 1,4,5-trisphosphate receptors. Nat Commun. 2017;8:15732. 10.1038/ncomms15732 PMC5472764 28598420

[21] Yao Y, Huang W, Li X, Li X, Qian J, Han H (2018). Tespa1 Deficiency Dampens Thymus-Dependent B-Cell Activation and Attenuates Collagen-Induced Arthritis in Mice. Front Immunol.

[22] Wang D, Zheng M, Qiu Y, Guo C, Ji J, Lei L (2014). Tespa1 negatively regulates FcepsilonRI-mediated signaling and the mast cell-mediated allergic response. J Exp Med.

[23] Yang R, Wang G, Li L, He H, Zheng M, Lu L (2020). Tespa1 plays a role in the modulation of airway hyperreactivity through the IL-4/STAT6 pathway. J Transl Med.

[24] Lu Y, Zhang Z, Wang S, Qi Y, Chen F, Xu Y (2022). Srebf1c preserves hematopoietic stem cell function and survival as a switch of mitochondrial metabolism. Stem Cell Rep.

[25] Zhang Z, Lu Y, Qi Y, Xu Y, Wang S, Chen F (2022). CDK19 regulates the proliferation of hematopoietic stem cells and acute myeloid leukemia cells by suppressing p53-mediated transcription of p21. Leukemia..

[26] Chu Y, Chen Y, Guo H, Li M, Wang B, Shi D (2020). SUV39H1 regulates the progression of MLL-AF9-induced acute myeloid leukemia. Oncogene..

[27] Sheng Y, Ma R, Yu C, Wu Q, Zhang S, Paulsen K (2021). Role of c-Myc haploinsufficiency in the maintenance of HSCs in mice. Blood..

[28] Numata A, Kwok HS, Zhou QL, Li J, Tirado-Magallanes R, Angarica VE (2020). Lysine acetyltransferase Tip60 is required for hematopoietic stem cell maintenance. Blood..

[29] Cheng Y, Luo H, Izzo F, Pickering BF, Nguyen D, Myers R (2019). m(6)A RNA Methylation Maintains Hematopoietic Stem Cell Identity and Symmetric Commitment. Cell Rep.

[30] Chen Y, Sun XX, Sears RC, Dai MS (2019). Writing and erasing MYC ubiquitination and SUMOylation. Genes Dis.

[31] Choi J, Baek KH (2018). Cellular functions of stem cell factors mediated by the ubiquitin-proteasome system. Cell Mol Life Sci.

[32] Wolf DA, Zhou C, Wee S (2003). The COP9 signalosome: an assembly and maintenance platform for cullin ubiquitin ligases?. Nat Cell Biol.

[33] Chen J, Shin JH, Zhao R, Phan L, Wang H, Xue Y (2014). CSN6 drives carcinogenesis by positively regulating Myc stability. Nat Commun.

[34] Hu M, Lu Y, Wang S, Zhang Z, Qi Y, Chen N (2022). CD63 acts as a functional marker in maintaining hematopoietic stem cell quiescence through supporting TGFbeta signaling in mice. Cell Death Differ.

[35] Murphy MJ, Wilson A, Trumpp A (2005). More than just proliferation: Myc function in stem cells. Trends Cell Biol.

[36] Adhikary S, Eilers M (2005). Transcriptional regulation and transformation by Myc proteins. Nat Rev Mol Cell Biol.

[37] Wilson A, Murphy MJ, Oskarsson T, Kaloulis K, Bettess MD, Oser GM (2004). c-Myc controls the balance between hematopoietic stem cell self-renewal and differentiation. Genes Dev.

[38] Menendez-Gutierrez MP, Porcuna J, Nayak RC, Paredes A, Niu H, Nunez V, et al. Retinoid X receptor promotes hematopoietic stem cell fitness and quiescence and preserves hematopoietic homeostasis. Blood. 2023;141:592–608. 10.1182/blood.2022016832 PMC10082360 36347014

[39] Holmfeldt P, Ganuza M, Marathe H, He B, Hall T, Kang G (2016). Functional screen identifies regulators of murine hematopoietic stem cell repopulation. J Exp Med.

[40] Zhu N, Chen M, Eng R, DeJong J, Sinha AU, Rahnamay NF (2016). MLL-AF9- and HOXA9-mediated acute myeloid leukemia stem cell self-renewal requires JMJD1C. J Clin Invest.

[41] Freire PR, Conneely OM (2018). NR4A1 and NR4A3 restrict HSC proliferation via reciprocal regulation of C/EBPα and inflammatory signaling. Blood..

[42] Sirin O, Lukov GL, Mao R, Conneely OM, Goodell MA (2010). The orphan nuclear receptor Nurr1 restricts the proliferation of haematopoietic stem cells. Nat Cell Biol.

[43] Min IM, Pietramaggiori G, Kim FS, Passegue E, Stevenson KE, Wagers AJ (2008). The transcription factor EGR1 controls both the proliferation and localization of hematopoietic stem cells. Cell Stem Cell.

[44] Xue Y, Chen J, Choi HH, Phan L, Chou PC, Zhao R (2012). HER2-Akt signaling in regulating COP9 signalsome subunit 6 and p53. Cell Cycle.

[45] Choi HH, Zou S, Wu JL, Wang H, Phan L, Li K (2020). EGF Relays Signals to COP1 and Facilitates FOXO4 Degradation to Promote Tumorigenesis. Adv Sci.

[46] Hou J, Deng Q, Zhou J, Zou J, Zhang Y, Tan P (2017). CSN6 controls the proliferation and metastasis of glioblastoma by CHIP-mediated degradation of EGFR. Oncogene..

[47] Sinha S, Dwivedi TR, Yengkhom R, Bheemsetty VA, Abe T, Kiyonari H (2019). Asrij/OCIAD1 suppresses CSN5-mediated p53 degradation and maintains mouse hematopoietic stem cell quiescence. Blood..

[48] Jung JH, Jung DB, Kim H, Lee H, Kang SE, Srivastava SK (2018). Zinc finger protein 746 promotes colorectal cancer progression via c-Myc stability mediated by glycogen synthase kinase 3beta and F-box and WD repeat domain-containing 7. Oncogene..

[49] Poole CJ, Zheng W, Lee H, Young D, Lodh A, Chadli A, et al. Targeting the MYC Oncogene in Burkitt Lymphoma through HSP90 Inhibition. Cancers (Basel). 2018;10:448. 10.3390/cancers10110448 PMC6266960 30453475

[50] Shlush LI, Zandi S, Mitchell A, Chen WC, Brandwein JM, Gupta V (2014). Identification of pre-leukaemic haematopoietic stem cells in acute leukaemia. Nature..

[51] Zhang L, Li J, Xu H, Shao X, Fu L, Hou Y (2020). Myc-Miz1 signaling promotes self-renewal of leukemia stem cells by repressing Cebp alpha and Cebp delta. Blood..

[52] Bill M, Papaioannou D, Karunasiri M, Kohlschmidt J, Pepe F, Walker CJ (2019). Expression and functional relevance of long non-coding RNAs in acute myeloid leukemia stem cells. Leukemia..

[53] Wang GG, Pasillas MP, Kamps MP (2005). Meis1 programs transcription of FLT3 and cancer stem cell character, using a mechanism that requires interaction with Pbx and a novel function of the Meis1 C-terminus. Blood..

[54] Kharas MG, Lengner CJ, Al-Shahrour F, Bullinger L, Ball B, Zaidi S (2010). Musashi-2 regulates normal hematopoiesis and promotes aggressive myeloid leukemia. Nat Med.

[55] Faber J, Krivtsov A, Stubbs M, Wright R, Davis T, van den Heuvel-Eibrink M (2009). HOXA9 is required for survival in human MLL-rearranged acute leukemias. Blood..

[56] Schmoellerl J, Barbosa IAM, Minnich M, Andersch F, Smeenk L, Havermans M (2023). EVI1 drives leukemogenesis through aberrant ERG activation. Blood..

[57] Cerami E, Gao J, Dogrusoz U, Gross BE, Sumer SO, Aksoy BA (2012). The cBio Cancer Genomics Portal: An Open Platform for Exploring Multidimensional Cancer Genomics Data. Cancer Disco.

[58] Tyner JW, Tognon CE, Bottomly D, Wilmot B, Kurtz SE, Savage SL (2018). Functional genomic landscape of acute myeloid leukaemia. Nature..

[59] Lechman ER, Gentner B, Ng SW, Schoof EM, van Galen P, Kennedy JA (2016). miR-126 Regulates Distinct Self-Renewal Outcomes in Normal and Malignant Hematopoietic Stem Cells. Cancer Cell.

[60] Sheng Y, Yu C, Liu Y, Hu C, Ma R, Lu X (2020). FOXM1 regulates leukemia stem cell quiescence and survival in MLL-rearranged AML. Nat Commun.

