# Supplementary material for: Tespa1 facilitates hematopoietic and leukemic stem cell maintenance by restricting c-Myc degradation
Source: Leukemia. 2023 Mar 30;37(5):1039–47. doi: 10.1038/s41375-023-01880-6 (PMC10169665; doi:10.1038/s41375-023-01880-6)
Supplement: Supplementary file 1 — Supplementary Information [file 41375_2023_1880_MOESM1_ESM.pdf]

Supplementary methods

Cell lines.

Human AML cell lines (HEL, MOLM-13, U937, THP-1 and NB4) were cultured in RPMI-1640 (Hyclone, South Logan, UT, USA) with 10% fetal bovine serum (FBS; Hyclone). Human stromal cells (HS-5) and HEK293T were cultured in DMEM (Hyclone) containing 10% FBS (Hyclone). All cell lines were obtained from Procell Life Science&Technology (Wuhan, China) or Beyotime (Shanghai, China) with authentication and maintained at 37°C with 5% CO2.

Primary human AML cell culture.

Primary human AML cells were cultured in StemSpan SFEM (Stem Cell Technologies, Vancouver, BC, Canada) supplemented with 20 ng/ml rhIL-3 (PeproTech), 100 ng/ml rhFlt3L (PeproTech), 50 ng/ml rhTPO (PeproTech), 100 ng/ml rhSCF (PeproTech), 1% penicillin–streptomycin (Beyotime) and 2 mM glutamine (Sigma, St. Louis, MO, USA). Cells were maintained at 37°C with 5% CO2.

Stress treatments.

For 5-FU challenge, mice were injected intraperitoneally (i.p.) with a single dose of 150 mg/kg 5-FU (Sigma) and analyzed by flow cytometry at day 9 after injection. For sublethal irradiation, mice were exposed to 5.0 Gy total body irradiation at Irradiation Center of Third Military Medical University and tested by flow cytometry at day 13 after IR.

Transplantation assays.

The competitive transplantation assays were conducted by intravenously transplanting

BM cells (1×10^6) or sorted LT-HSCs (1×10^3) from *Tespa1*$^{+/+}$ and *Tespa1*$^{-/-}$ mice, together with CD45.1$^+$ BM cells (1×10^6), into lethally irradiated (10.0 Gy) CD45.1 recipients. The secondary transplantation assays were performed by injecting BM cells (1×10^6) from primary recipients at 16 weeks into new lethally irradiated (10.0 Gy) CD45.1 recipients. PB chimerism was evaluated every 4 weeks and BM chimerism was analyzed at 16 weeks after transplantation. For reciprocal transplantation, experiments were performed as we previously reported. For homing assay, sorted LSKs (5×10^4) from *Tespa1*$^{+/+}$ and *Tespa1*$^{-/-}$ mice were transplanted into CD45.1 recipients receiving lethal irradiation (10.0 Gy) and homing efficiency was analyzed by flow cytometry at 16 hours after transplantation.

Duolink proximity ligation assay (PLA).

Duolink PLA was conducted using the Duolink In Situ Red Starter Kit (Sigma) following the manufacturer's instructions. In brief, LSKs sorted from *Tespa1*$^{+/+}$ and *Tespa1*$^{-/-}$ mice after 5-FU or IR treatment were spun onto slides. Cells were fixed, permeabilized, blocked and then incubated with anti-Tespa1 (Invitrogen, Carlsbad, CA, USA) and anti-CSN6 (Santa Cruz, Dallas, TX, USA) at 4°C overnight. After washing twice, slides were stained with PLA probe at 37°C for 60 min, ligated with ligase at 37°C for 30 min, and amplified with polymerase at 37°C for 100 min. After staining with 4',6-diamidino-2-phenylindole (DAPI; Sigma), slides were sealed using medium and imaged using a confocal microscope (LSM780; Carl Zeiss, Jena, Germany). Detailed information of antibodies is provided in Supplementary Table S1.

Immunofluorescence.

LSKs were sorted from *Tespa1*$^{+/+}$ and *Tespa1*$^{-/-}$ mice after 5-FU or IR treatment and spun onto slides. Then, cells were fixed with 4% paraformaldehyde for 15 min, permeabilized with 0.5% Triton X-100 for 15 min. After blocking, cells were incubated with anti-Tespa1 (Invitrogen) and anti-CSN6 (Santa Cruz) antibodies at 4°C overnight, and then stained with fluorochrome-conjugated secondary antibodies (Invitrogen) at room temperature (RT) for 60 min. Finally, sample imaging was performed using a Zeiss LSM780 confocal microscope (Carl Zeiss). Detailed information of antibodies is provided in Supplementary Table S1.

Western blot and co-immunoprecipitation analysis.

For Western blot analysis, cells were washed with phosphate buffered saline (PBS) and then lysed by RIPA buffer (Beyotime). Cell lysates were fractionated using 10% SDS-PAGE (EpiZyme, Shanghai, China) and transferred onto the PVDF membranes (Merck Millipore, Burlington, MA, USA). After blocking, the membranes were incubated with anti-Tespa1 (Invitrogen) or anti-GAPDH (Beyotime) overnight 4°C, followed by incubation with HRP-conjugated secondary antibodies (Beyotime) at 37°C for 1 hour. Finally, all bands were detected using Pierce ECL Western Blotting Substrate (Thermo Fisher Scientific, Waltham, MA, USA) or BeyoECL Star (Beyotime).

For immunoprecipitation analysis, Lin^- $c\text{-}Kit^+$ cells sorted from *Tespa1*$^{+/+}$ and *Tespa1*$^{-/-}$ mice upon 5-FU and IR exposure were extracted by RIPA buffer (Beyotime). Then, cell lysates were incubated with indicated antibodies pre-coupled to protein G magnetic beads (Invitrogen). After washing and eluting, proteins were collected for Western blot analysis. The host species of antibodies used for immunoprecipitation

were different from those of primary antibodies used for Western blot to avoid the interference from the heavy chain and light chain. Detailed information of antibodies is provided in Supplementary Table S1.

RNA extraction and qPCR analysis.

Total RNA extraction was performed using the RNA queous-Micro Kit (Invitrogen) according to the manufacturer's protocols. RNA was reversely transcribed to cDNA using the PrimeScript RT Reagent Kit (TaKaRa, Tokyo, Japan) and then subjected to PCR analysis using the SYBR Premix Ex Taq II Kit (TaKaRa). The relative expression of each gene was normalized to *Gapdh* and determined by $2^{-\Delta\Delta Ct}$ methods. Detailed information of primer sequences is provided in Supplementary Table S2.

RNA-seq and data analysis.

RNA was purified from *Tespa1*$^{+/+}$ and *Tespa1*$^{-/-}$ LSKs at day 9 after 5-FU treatment or at day 13 upon IR exposure. Subsequently, RNA was submitted to Sinotech Genomics Co., Ltd (Shanghai, China) to perform RNA-seq. Fold change >1.5 and P value < 0.05 was used to define differential expressed genes. Heatmaps and volcano plots were visualized using R package ComplexHeatmap and EnhancedVolcano, respectively. GSEA was conducted using GSEA_4.0.3 software.

Serial replating assay.

Pre-leukemic cells were seeded in MethoCultTM M3434 (Stem Cell Technologies) at the density of 1×10^3 cells per well. After incubation at 37°C for 7 days, colonies were counted and harvested for replating in a new medium for another 7 days.

Public database analysis.

The expression of TESPA1 in AML samples and normal human controls were analyzed using TCGA database and GEO dataset (GSE300029). The scRNA-seq data of AML cells were obtained from the Atlas of blood cells (http://abc.sklehabc.com/) under the accession no. EGAD00001008185. ROC curve was calculated using R package pROC. The data about TESPA1 expression in AML patients with remission or relapse were obtained from the Vizome database (http://www.vizome.org/). Kaplan-Meier survival analysis of AML patients was performed using the data from GEO database (GSE12417).

Code availability.

The code used for R analysis are available from the corresponding author on reasonable request.

Supplementary Tables

Table S1. Antibodies used in flow cytometry, duolink proximity ligation, immunofluorescence, Western blot and immunoprecipitation

Table S2. Primer sequences for mRNA expression and shRNA sequences for TESPA1 knockdown

Supplementary Figure S1

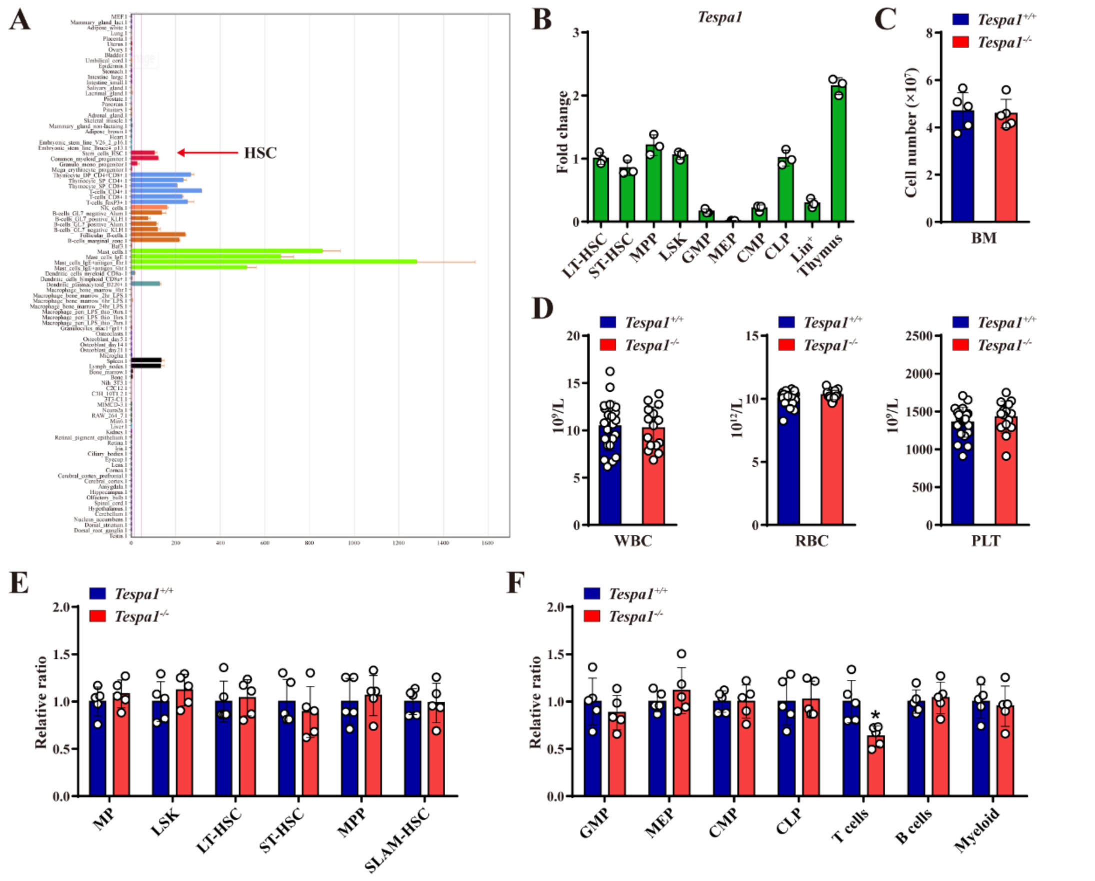

Supplementary Figure S1. The effect of Thymocyte-expressed, positive selection-associated 1 (Tespa1) deletion on hematopoiesis in mice under steady-state.

(A) Tespa1 expression profile obtained from the BioGPS dataset (http://biogps.org/). (B) Relative expression levels of Tespa1 mRNA in different hematopoietic cell subsets sorted from normal wild-type (WT) mice were detected by quantitative real-time PCR (qPCR) (n = 3). Long-term HSC (LT-HSC), $Lin^- Sca1^+ c\text{-}Kit^+ CD34^- Flk2^-$; short-term HSC (ST-HSC), $Lin^- Sca1^+ c\text{-}Kit^+ CD34^+ Flk2^-$; multipotent progenitor (MPP), $Lin^- Sca1^+ c\text{-}Kit^+ CD34^+ Flk2^+$; LSK, $Lin^- Sca1^+ c\text{-}Kit^+$; granulocyte/macrophage progenitor (GMP), $Lin^- CD127^- Sca1^- c\text{-}Kit^+ CD16/32^+ CD34^+$; megakaryocyte/erythroid progenitor (MEP), $Lin^- CD127^- Sca1^- c\text{-}Kit^+ CD16/32^- CD34^-$; common myeloid

progenitor (CMP), Lin^- $CD127^-$ $Sca1^-$ $c\text{-}Kit^+$ $CD16/32^-$ $CD34^+$; common lymphoid progenitor (CLP), Lin^- $CD127^+$ $Sca1^{med}$ $c\text{-}Kit^{med}$; Lin^+, $Lineage^+$. (C) Bone marrow (BM) cellularity of *Tespa1*$^{+/+}$ and *Tespa1*$^{-/-}$ mice at steady-state (n = 5). (D) Peripheral blood (PB) counts in *Tespa1*$^{+/+}$ and *Tespa1*$^{-/-}$ mice at a steady state (n = 15-22). WBC, white blood cell. RBC, red blood cell. PLT, platelet. (E) Flow cytometric analysis of the percentages of myeloid progenitors (MPs), LSKs, LT-HSCs, ST-HSCs, MPPs, and signaling lymphocytic activation molecule (SLAM)-HSCs (Lin^- $Sca1^+$ $c\text{-}Kit^+$ $CD150^+$ $CD48^-$) in the BM of *Tespa1*$^{+/+}$ and *Tespa1*$^{-/-}$ mice at steady-state (n = 5). (F) The relative percentages of lineage-committed progenitor cells (GMPs, MEPs, CMPs and CLPs) and mature cells (T cells, B cells and myeloid cells) in the BM of *Tespa1*$^{+/+}$ and *Tespa1*$^{-/-}$ mice at steady-state (n = 5). *$P < 0.05$.

Supplementary Figure S2

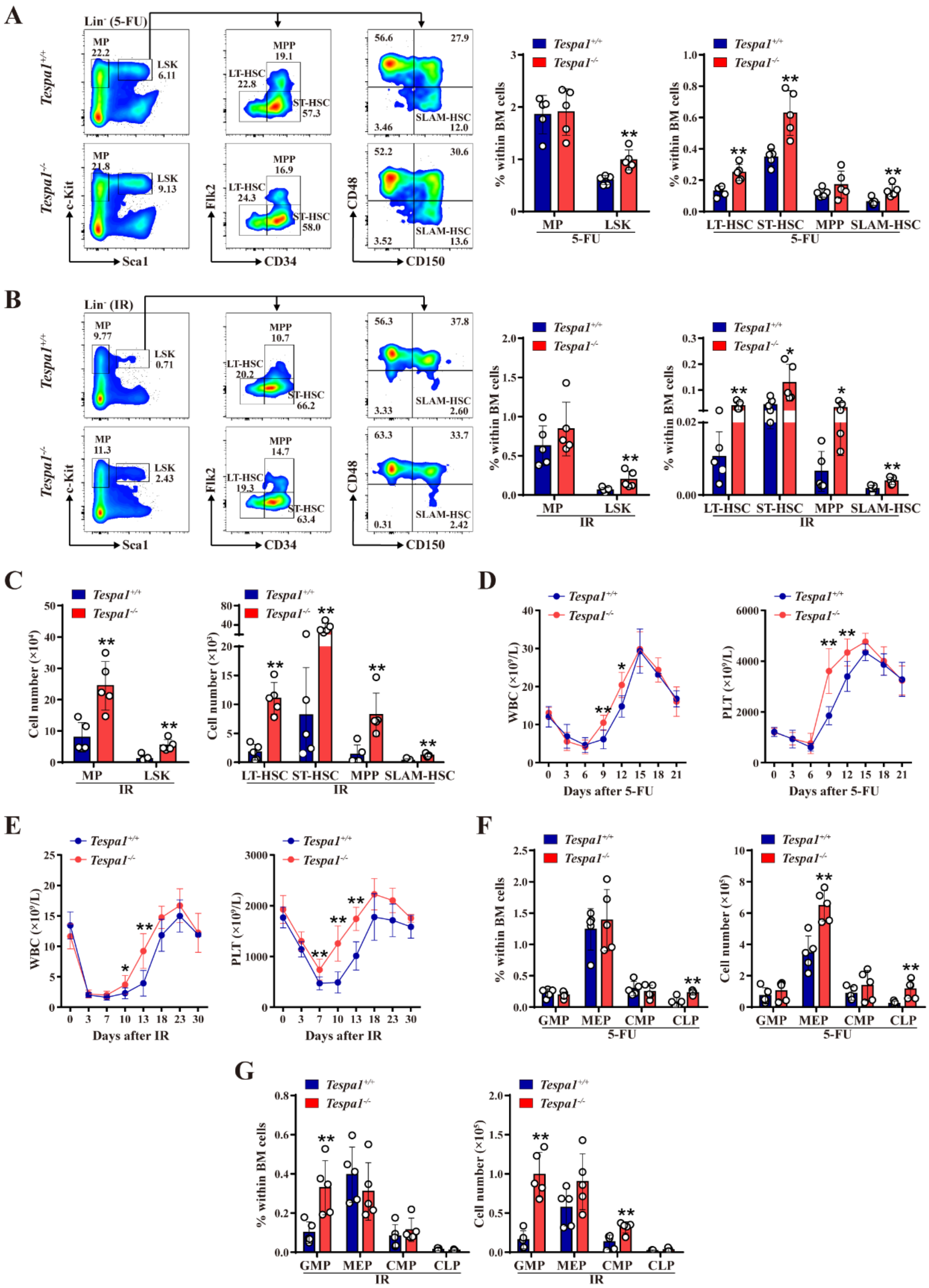

Supplementary Figure S2. Tespa1 deficiency promotes hematopoietic recovery in mice after stresses.

(A, B) Flow cytometric analysis of the percentages of indicated populations in the BM

of *Tespa1*$^{+/+}$ and *Tespa1*$^{-/-}$ mice (A) at day 9 following 5-fluorouracil (5-FU) injection or (B) at day 13 after irradiation (IR) exposure (n = 5). (C) The numbers (two femurs and tibias) of indicated populations in the BM of *Tespa1*$^{+/+}$ and *Tespa1*$^{-/-}$ mice at day 13 after IR exposure (n = 5). (D, E) The counts of WBC and PLT in the PB of *Tespa1*$^{+/+}$ and *Tespa1*$^{-/-}$ mice at the indicated time points following (D) 5-FU injection or (E) IR exposure (n = 5-11). (F, G) The percentages and numbers (two femurs and tibias) of lineage-committed progenitor cells in *Tespa1*$^{+/+}$ and *Tespa1*$^{-/-}$ BM (F) at day 9 following 5-FU injection or (G) at day 13 after IR exposure (n = 5). *P < 0.05, **P < 0.01.

Supplementary Figure S3

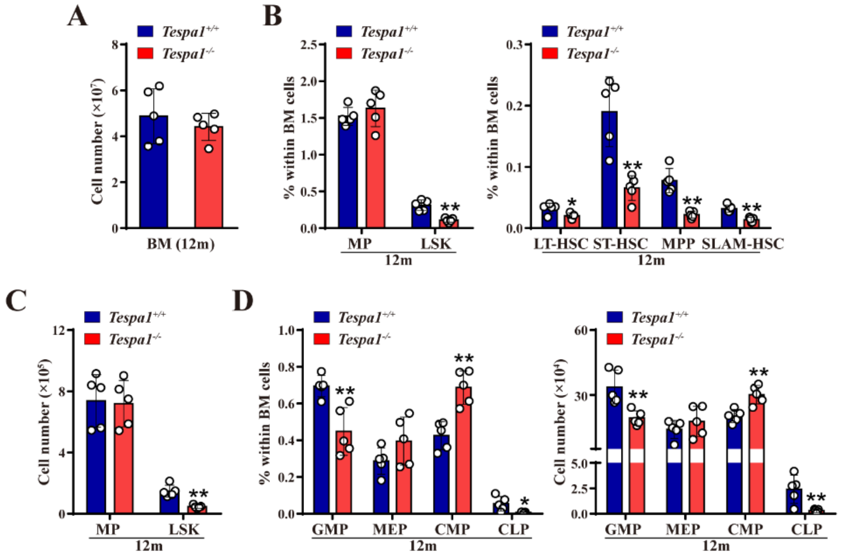

Supplementary Figure S3. Tespa1 ablation leads to reduced HSC pools in middle aged mice.

(A) The numbers (two femurs and tibias) of BM cells of 12-month (m)-old *Tespa1*$^{+/+}$ and *Tespa1*$^{-/-}$ mice (n = 5). (B) Flow cytometric analysis of the percentages of hematopoietic stem and progenitor cells (HSPCs) in the BM of 12-month-old *Tespa1*$^{+/+}$ and *Tespa1*$^{-/-}$ mice (n = 5). (C) The numbers (two femurs and tibias) of indicated populations in the BM of 12-month (m)-old *Tespa1*$^{+/+}$ and *Tespa1*$^{-/-}$ mice (n = 5). (D) Flow cytometric analysis of the percentages and numbers (two femurs and tibias) of lineage-committed progenitor cells in the BM of 12-month-old *Tespa1*$^{+/+}$ and *Tespa1*$^{-/-}$ mice (n = 5). $^{*}P < 0.05$, $^{**}P < 0.01$.

Supplementary Figure S4

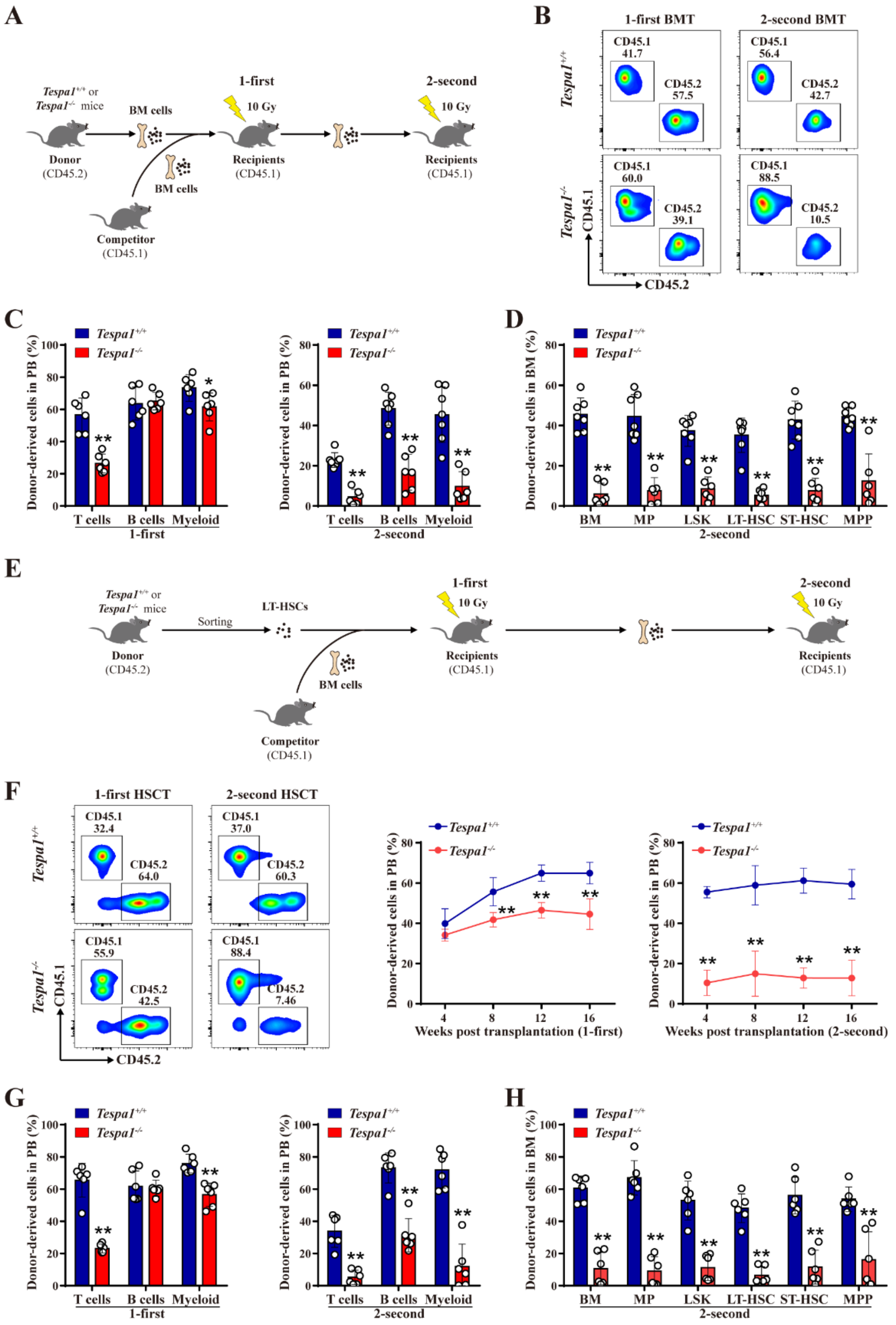

Supplementary Figure S4. Tespa1 maintains long-term HSC self-renewal ability.

(A) Schematic for competitive BM transplantation (BMT) experiment. (B) Representative flow cytometric plots showing the percentages of donor-derived cells in

recipients' PB at the indicated time points after first and second BMT. (C) The percentages of donor-derived T cells, B cells, and myeloid cells in PB of recipient mice at 16 weeks after (left) first and (right) second BMT (n = 6-7). (D) The percentages of donor-derived BM cells, MPs, LSKs, LT-HSCs, ST-HSCs, and MPPs in recipient mice at 16 weeks after second BMT (n = 6-7). (E) Schematic for competitive HSC transplantation (HSCT) experiment. (F) The percentages of donor-derived cells in recipients' PB at the indicated time points in (left) first and (right) second HSCT (n = 6-7). Representative flow cytometric plots at 16 weeks after first and second HSCT are shown in the left. (G) The percentages of donor-derived T cells, B cells, and myeloid cells in PB of recipient mice at 16 weeks after (left) first and (right) second HSCT (n = 6). (H) The percentages of donor-derived BM cells, MPs, LSKs, LT-HSCs, ST-HSCs, and MPPs in recipient mice at 16 weeks after second HSCT (n = 6). *P < 0.05, **P < 0.01.

Supplementary Figure S5

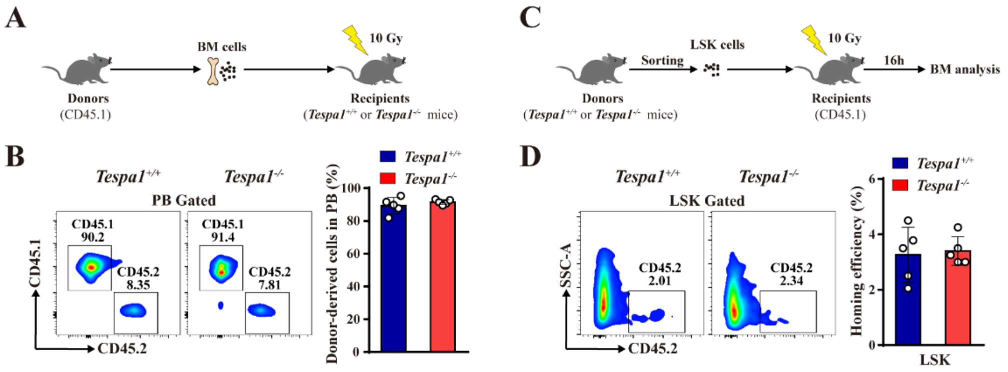

Supplementary Figure S5. Tespa1 regulates HSC biology in an intrinsic manner.

(A, B) Reciprocal BMT experiments of *Tespa1*$^{+/+}$ and *Tespa1*$^{-/-}$ mice (n = 5). (C, D) Homing assays of LSKs from *Tespa1*$^{+/+}$ and *Tespa1*$^{-/-}$ mice (n = 5).

Supplementary Figure S6

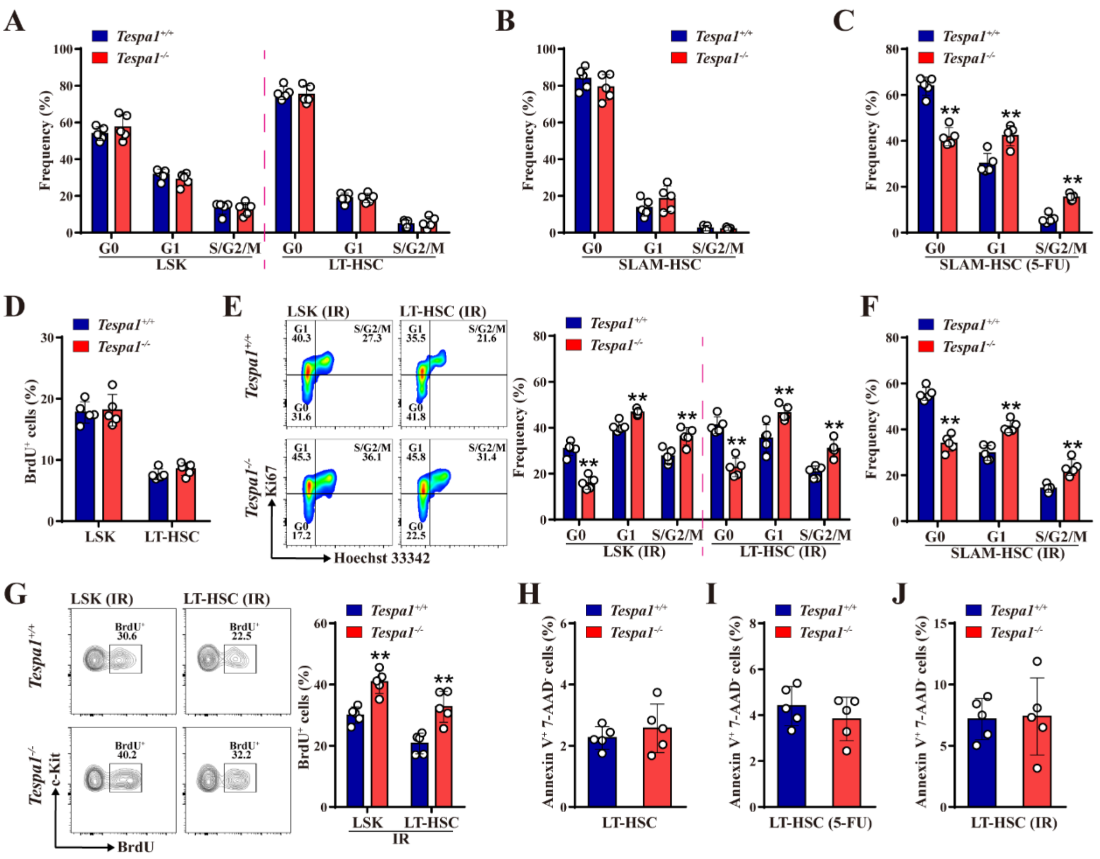

Supplementary Figure S6. Loss of Tespa1 promotes HSC proliferation after stress but not at steady-state.

(A, B) Cell cycle analysis of (A) LSKs, LT-HSCs, and (B) SLAM-HSCs in the BM of *Tespa1*$^{+/+}$ and *Tespa1*$^{-/-}$ mice at steady-state (n = 5). (C) Cell cycle analysis of SLAM-HSCs in the BM of *Tespa1*$^{+/+}$ and *Tespa1*$^{-/-}$ mice at day 9 after 5-FU injection (n = 5). (D) The proportion of bromodeoxyuridine (BrdU)$^{+}$ cells in LSKs and LT-HSCs from *Tespa1*$^{+/+}$ and *Tespa1*$^{-/-}$ BM at steady-state (n = 5). (E) Flow cytometric analysis of the cell cycle of LSKs and LT-HSCs in the BM of *Tespa1*$^{+/+}$ and *Tespa1*$^{-/-}$ mice at day 13 after IR exposure (n = 5). (F) Cell cycle analysis of SLAM-HSCs in the BM of *Tespa1*$^{+/+}$ and *Tespa1*$^{-/-}$ mice at day 13 after IR exposure (n = 5). (G) Flow cytometric analysis of the proportion of BrdU^{+} cells in LSKs and LT-HSCs from *Tespa1*$^{+/+}$ and

Tespa1$^{-/-}$ mice at day 13 after IR exposure (n = 5). (H-J) Flow cytometric analysis of the apoptosis in LT-HSCs from *Tespa1*$^{+/+}$ and *Tespa1*$^{-/-}$ mice (H) at steady-state, (I) at day 9 following 5-FU injection, and (J) at day 13 after IR exposure (n = 5). **P < 0.01.

Supplementary Figure S7

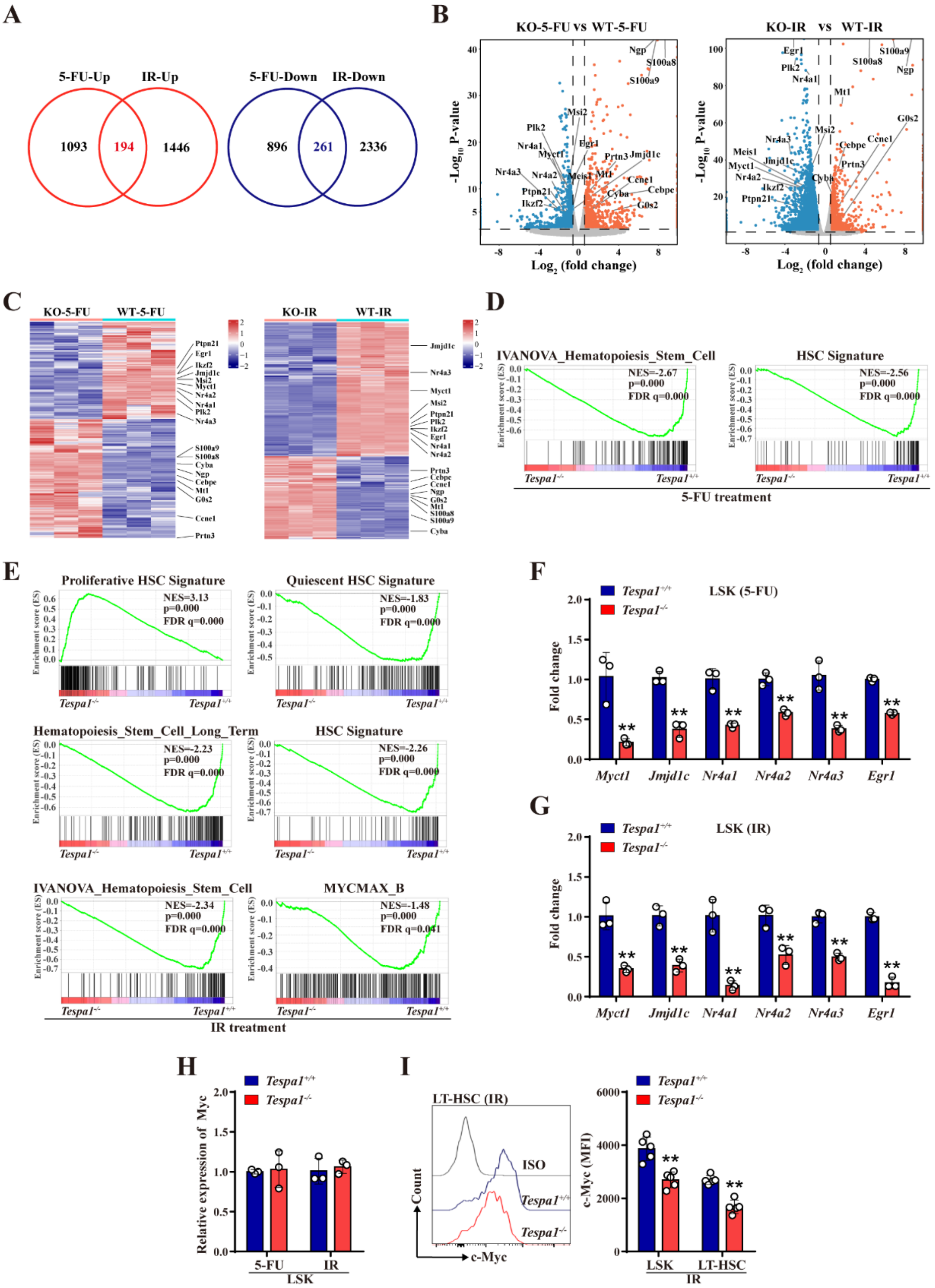

Supplementary Figure S7. Tespa1 deficiency reduces stemness-related signatures and c-Myc target gene expression in HSCs upon stress.

(A) Transcriptomes profiling of LSKs sorted from *Tespa1*$^{+/+}$ and *Tespa1*$^{-/-}$ mice at day

9 following 5-FU injection or at day 13 after IR exposure, respectively. Red-colored box represents upregulated genes and blue-colored box represents downregulated genes in LSKs after *Tespa1* knockout (KO). (B) The volcano plots of upregulated genes or downregulated genes by 1.5-fold or more in *Tespa1*$^{-/-}$ (KO) LSKs in relative to *Tespa1*$^{+/+}$ (WT) LSKs following (left) 5-FU and (right) IR treatment ($p < 0.05$). (C) The heatmap plots of upregulated genes or downregulated genes by 1.5-fold or more in *Tespa1*$^{-/-}$ (KO) LSKs in relative to *Tespa1*$^{+/+}$ (WT) LSKs following (left) 5-FU and (right) IR treatment ($p < 0.05$). Representative common differentially expressed genes are indicated. (D) Gene set enrichment analysis (GSEA) of RNA-sequencing (RNA-seq) data with HSC-related gene sets after 5-FU treatment. (E) GSEA of RNA-seq data with HSC-related gene sets and MYC-related gene set after IR exposure. (F, G) qPCR analysis of the mRNA expression of indicated genes in LSKs isolated from *Tespa1*$^{+/+}$ and *Tespa1*$^{-/-}$ mice (F) at day 9 following 5-FU injection or (G) at day 13 after IR exposure (n = 3). (H) qPCR analysis of c-Myc mRNA expression in LSKs from *Tespa1*$^{+/+}$ and *Tespa1*$^{-/-}$ mice at day 9 following 5-FU injection or at day 13 after IR exposure (n = 3). (I) Flow cytometric analysis of c-Myc protein expression in LSKs and LT-HSCs from *Tespa1*$^{+/+}$ and *Tespa1*$^{-/-}$ mice at day 13 after IR exposure (n = 5). **P < 0.01.

Supplementary Figure S8

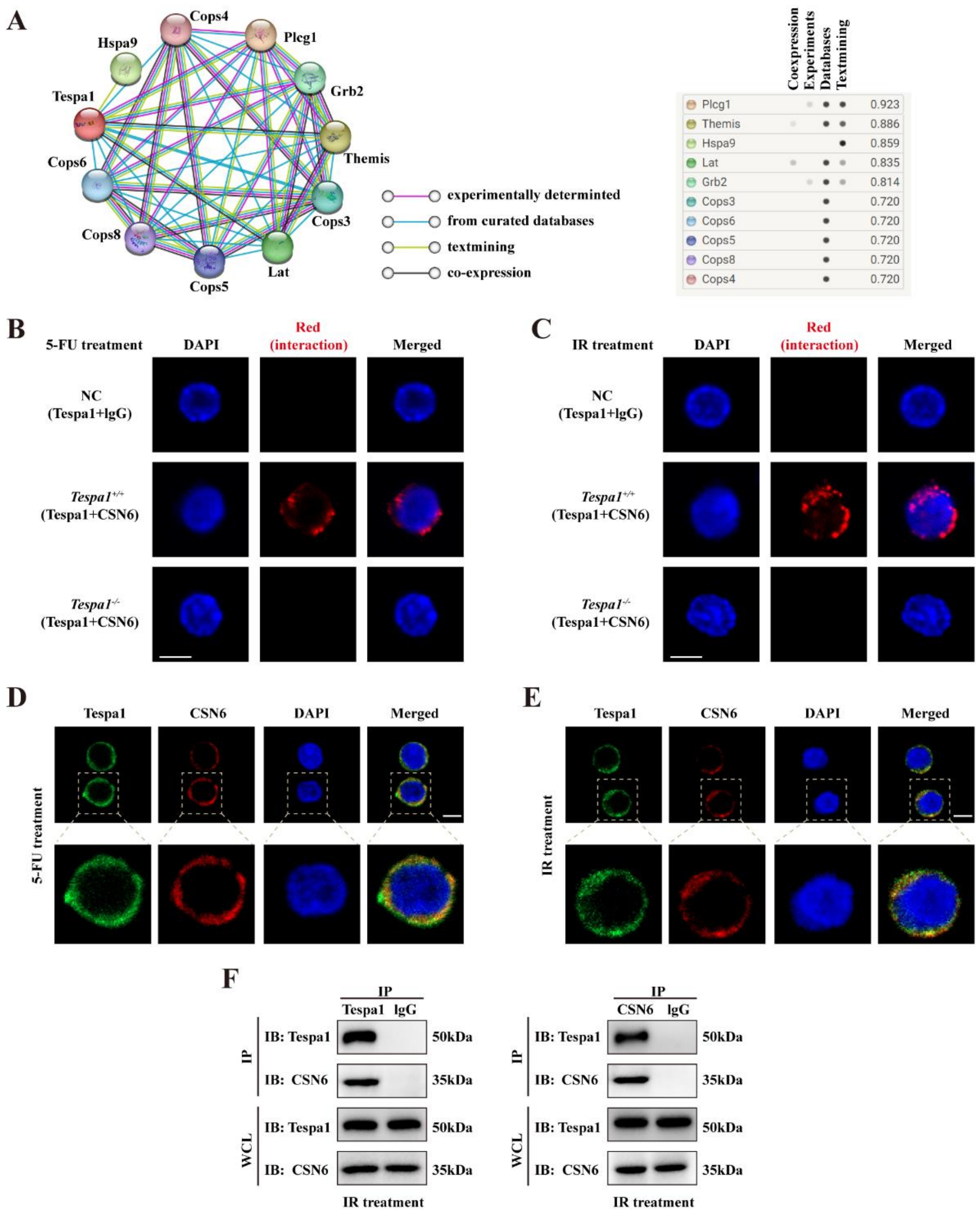

Supplementary Figure S8. Tespa1 interacts with CSN subunit 6 (CSN6) in HSCs. (A) The potential Tespa1 interacting factors in STRING database (http://string-db.org). (B, C) *In situ* proximity ligation assays for analysis of the interaction of Tespa1 and CSN6 in LSKs sorted from the BM of mice (B) at day 9 following 5-FU injection or (C) at day 13 after IR treatment. As a negative control, proximity ligation was

performed using a rabbit anti-Tespa1 antibody and a mouse IgG. The scale bar represents 5 μm. (D, E) Immunofluorescence analysis of the colocalization of Tespa1 and CSN6 in LSKs sorted from normal mice (D) at day 9 following 5-FU injection or (E) at day 13 after IR treatment. The scale bar indicates 5 μm. (F) Co-immunoprecipitation analysis of Tespa1 and CSN6 interaction in Lin^- $c\text{-}Kit^+$ cells purified from the BM of mice at day 13 after IR treatment. IgG antibody was used as the negative control.

Supplementary Figure S9

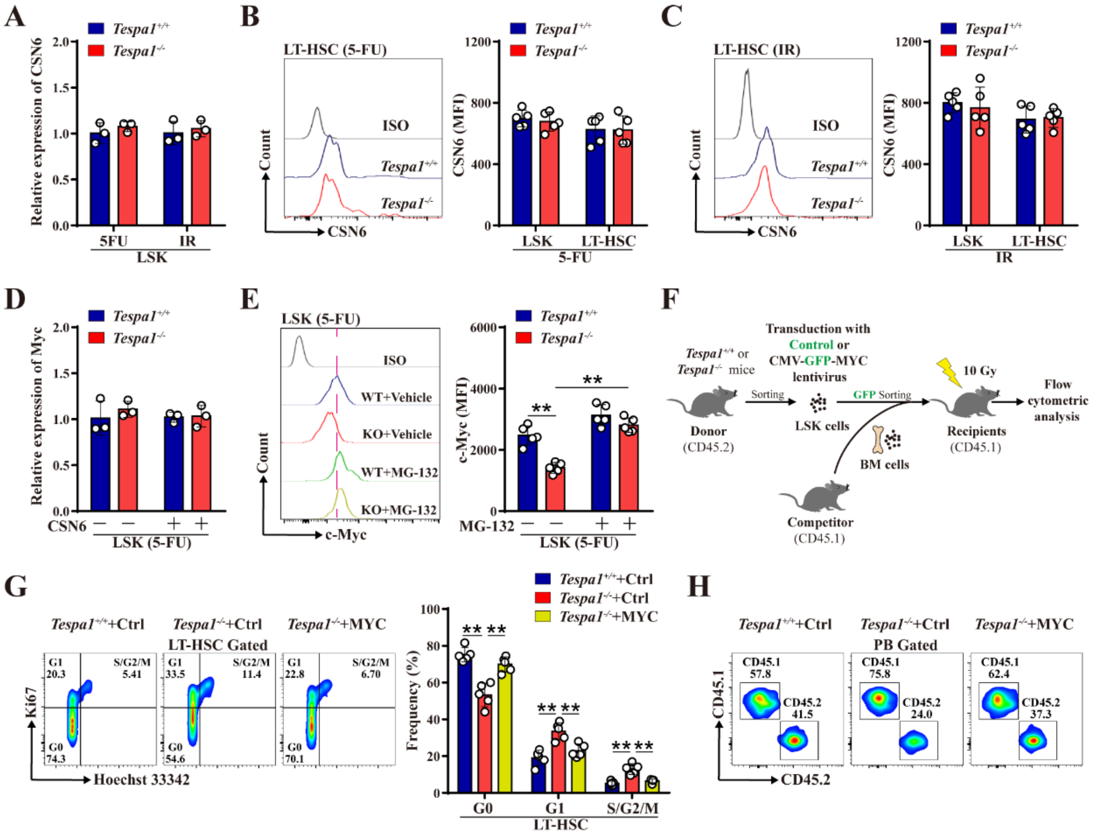

Supplementary Figure S9. Myc downregulation is responsible for the deficiency of Tespa1-null HSCs.

(A) qPCR analysis of CSN6 mRNA expression in LSKs sorted from *Tespa1*$^{+/+}$ and *Tespa1*$^{-/-}$ mice at day 9 following 5-FU injection or at day 13 after IR treatment (n = 3). (B, C) Flow cytometric analysis of CSN6 protein expression in LSKs and LT-HSCs from *Tespa1*$^{+/+}$ and *Tespa1*$^{-/-}$ mice (B) at day 9 following 5-FU injection or (C) at day 13 after IR treatment (n = 5). (D) LSKs from the BM of *Tespa1*$^{+/+}$ and *Tespa1*$^{-/-}$ mice at day 9 post 5-FU injection were transduced with control (Ctrl) or CSN6 overexpression lentivirus. Then, c-Myc mRNA expression in LSKs was analyzed by qPCR after transduction (n = 3). (E) LSKs were sorted from the BM of *Tespa1*$^{+/+}$ and *Tespa1*$^{-/-}$ mice at day 9 post 5-FU injection. Flow cytometric analysis of c-Myc protein

expression in LSKs after culture with or without MG-132 (50 μg/ml) for 6 hours (n = 5). (F-H) LSKs from *Tespa1*$^{+/+}$ and *Tespa1*$^{-/-}$ BM were transduced with control (Ctrl) or c-Myc overexpression lentivirus. Green fluorescent protein (GFP)$^{+}$ cells were then transplanted into CD45.1 recipient mice with CD45.1 competitor BM cells. (F) Schematic for experiment strategy. (G) Cell cycle distribution in donor-derived LT-HSCs at 16 weeks after transplantation (n = 5). Representative flow cytometric plots are shown in left. (H) Representative flow cytometric plots showing the percentage of donor-derived cells in PB of recipient mice at 16 weeks after transplantation. **P < 0.01.

Supplementary Figure S10

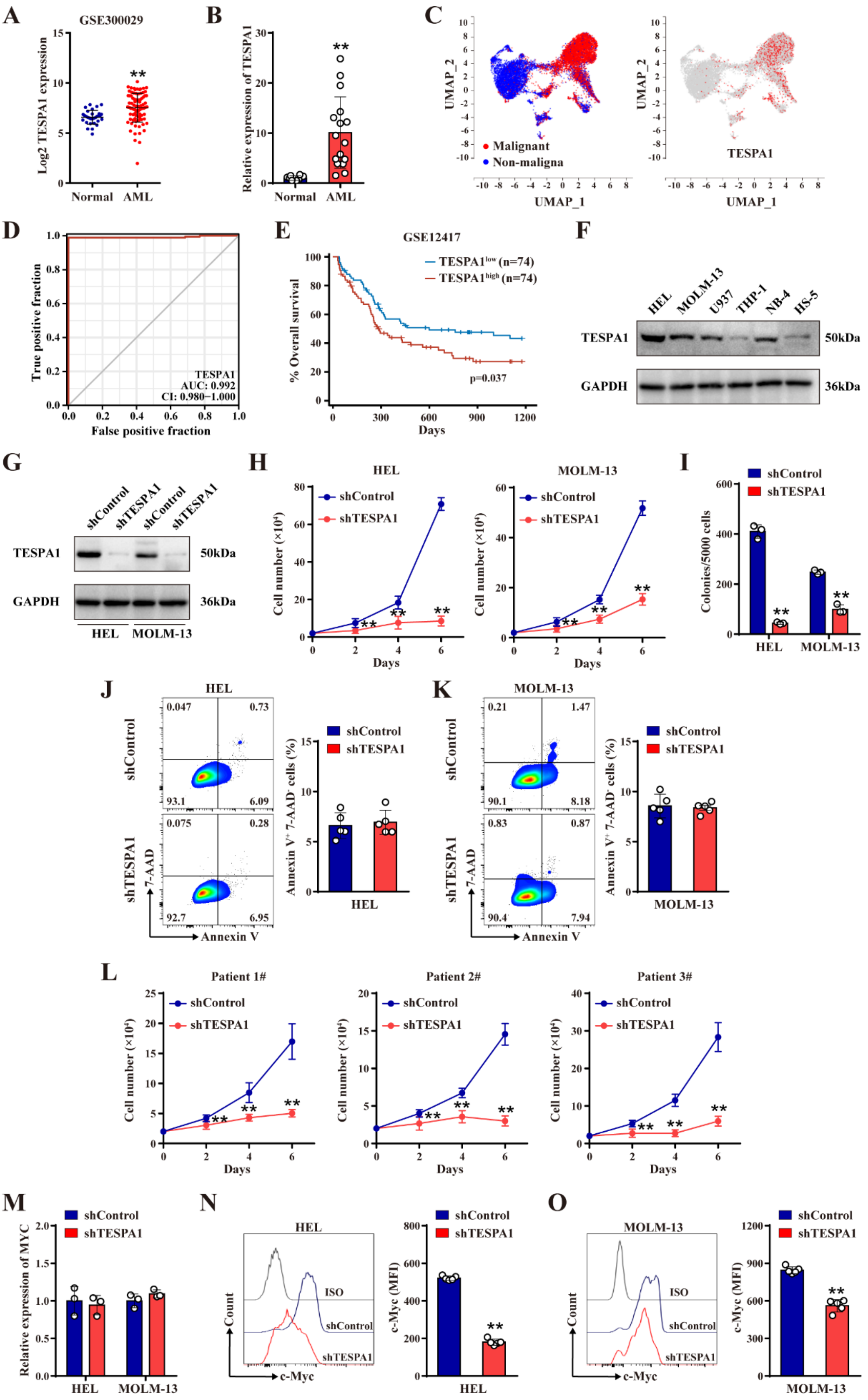

372

373 **Supplementary Figure S10. TESPA1 knockdown suppresses the growth of human**

acute myeloid leukemia (AML) cells.

(A) The expression of TESPA1 in AML patients and healthy donors. The data (90 AML patients and 31 healthy donors) were derived from Gene expression Ominibus (GEO) database (GSE300029). (B) qPCR analysis of TESPA1 expression in BM samples from 16 AML patients and 10 healthy donors. (C) Single-cell analysis of TESPA1 expression in the BM of AML patients. Data were derived from the European Genome-Phenome Archive (EGA) database (EGAD00001008185). (D) Receiver-operating characteristic (ROC) curve of the diagnostic performance of TESPA1 for AML. (E) Kaplan-Meier plots showing the overall survival of AML patients with high or low expression of TESPA1. The patients were divided into two groups based on the median expression levels of TESPA1. Data were obtained from GEO database (GSE12417). (F) Western blot analysis of TESPA1 protein expression in lysates from AML cell lines. (G) Western blot analysis of TESPA1 protein expression in HEL and MOLM-13 cells at 48 hours after transduction with lentivirus carrying shControl or shTESPA1. (H) The growth curves of HEL and MOLM-13 cells after TESPA1 knockdown ($n = 6$). (I) The colony formation assays of HEL and MOLM-13 cells after TESPA1 knockdown ($n = 3$). (J, K) Flow cytometric analysis of the apoptosis in (J) HEL and (K) MOLM-13 cells after TESPA1 knockdown ($n = 5$). (L) The growth curves of primary human AML cells after TESPA1 knockdown. (M) qPCR analysis of c-Myc mRNA expression in HEL and MOLM-13 cells after TESPA1 knockdown ($n = 3$). (N, O) Flow cytometric analysis of c-Myc protein expression in (N) HEL and (O) MOLM-13 cells after TESPA1 knockdown ($n = 5$). **$P < 0.01$.

Supplementary Figure S11

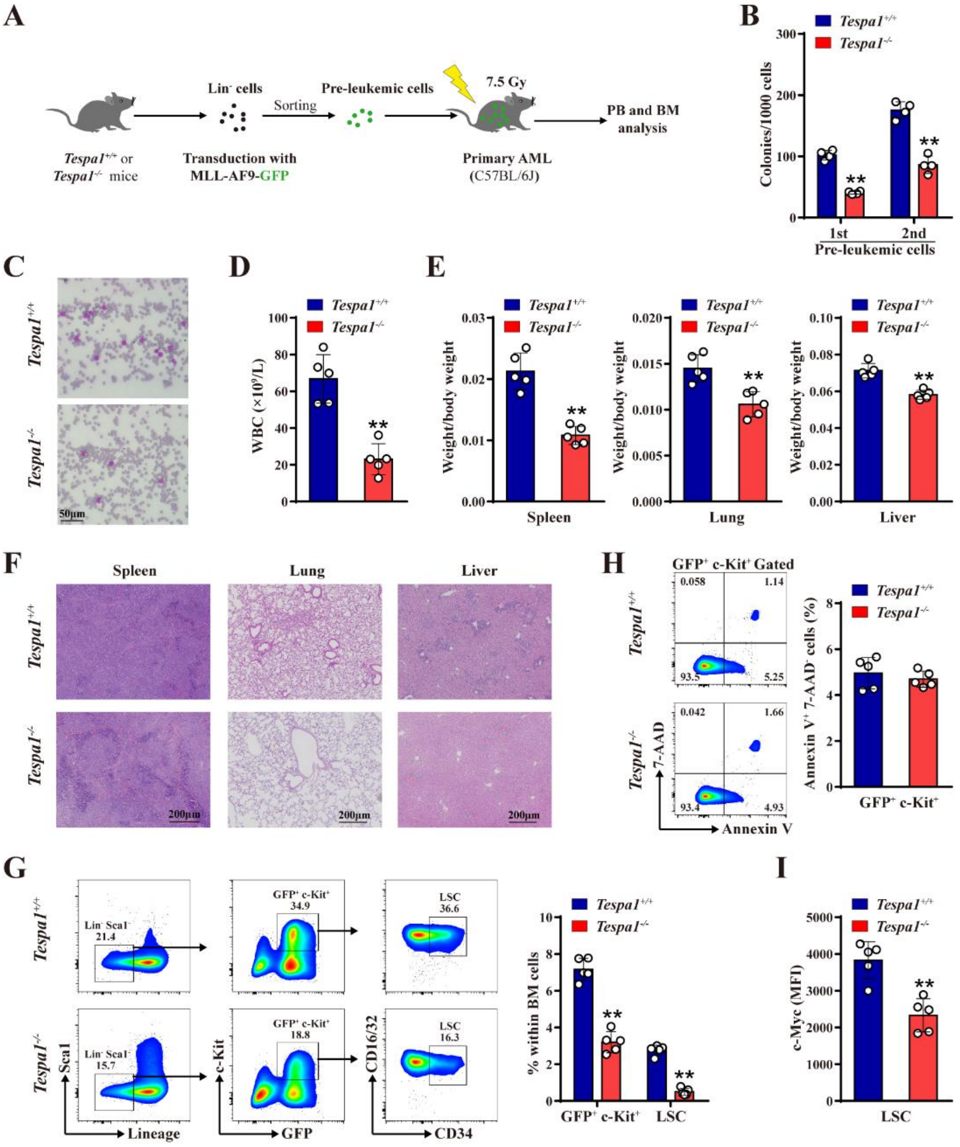

Supplementary Figure S11. Tespa1 knockout delays AML progression in mice.

(A) Schematic for MLL-AF9-induced murine AML model. (B) The colony formation assays of pre-leukemic (GFP^{+}) cells sorted from BM Lin^{-} cells after transduced with MLL-AF9-GFP retrovirus (n = 4). (C) Giemsa staining of PB smears of mice at 25 days after transplanted with *Tespa1*$^{+/+}$ and *Tespa1*$^{-/-}$ pre-leukemic cells. (D) The counts of WBC in the PB of mice at 25 days after transplanted with *Tespa1*$^{+/+}$ and *Tespa1*$^{-/-}$ pre-leukemic cells (n = 5). (E) Relative weights (weights/body weights) of the spleens,

lungs, and livers from recipients at day 25 after transplantation with *Tespa1*$^{+/+}$ and *Tespa1*$^{-/-}$ pre-leukemic cells (n = 5). (F) Hematoxylin-eosin staining of the spleens, lungs, and livers from primary AML mice at day 25 post-transplant. (G) Flow cytometric analysis of the percentages of GFP^{+} c-Kit^{+} cells and leukemia stem cells (LSCs) in the BM from recipients at day 25 after transplantation (n = 5). LSC, Lin^{-} Sca1^{-} GFP^{+} c-Kit^{+} CD34^{+} CD16/32^{+}. (H) Flow cytometric analysis of the apoptosis in GFP^{+} c-Kit^{+} cells from recipients at day 25 after transplantation (n = 5). (I) The expression levels of c-Myc protein in LSCs from the BM of primary AML mice at day 25 post-transplant (n =5). **$P < 0.01$.
